# Critical role of backbone coordination in the mRNA recognition by RNA induced silencing complex

**DOI:** 10.1038/s42003-021-02822-7

**Published:** 2021-11-30

**Authors:** Lizhe Zhu, Hanlun Jiang, Siqin Cao, Ilona Christy Unarta, Xin Gao, Xuhui Huang

**Affiliations:** 1 Warshel Institute for Computational Biology, School of Life and Health Sciences, The Chinese University of Hong Kong (Shenzhen), Shenzhen, Guangdong 518172 China; 2 Department of Chemistry, The Hong Kong University of Science and Technology, Clear Water Bay, Kowloon, Hong Kong; 3 Department of Chemical and Biological Engineering, The Hong Kong University of Science and Technology, Clear Water Bay, Kowloon, Hong Kong; 4 Department of Biochemistry, Institute for Protein Design, University of Washington, Seattle, WA 98195 USA; 5 Center of Systems Biology and Human Health, State Key Laboratory of Molecular Neuroscience, The Hong Kong University of Science and Technology, Clear Water Bay, Kowloon, Hong Kong; 6 Computational Bioscience Research Center, Computer, Electrical and Mathematical Sciences and Engineering Division, King Abdullah University of Science and Technology (KAUST), Thuwal, 23955-6900 Saudi Arabia

**Keywords:** Computational models, Computational biophysics

## Abstract

Despite its functional importance, the molecular mechanism underlying target mRNA recognition by Argonaute (Ago) remains largely elusive. Based on extensive all-atom molecular dynamics simulations, we constructed quasi-Markov State Model (qMSM) to reveal the dynamics during recognition at position 6-7 in the seed region of human Argonaute 2 (hAgo2). Interestingly, we found that the slowest mode of motion therein is not the gRNA-target base-pairing, but the coordination of the target phosphate groups with a set of positively charged residues of hAgo2. Moreover, the ability of Helix-7 to approach the PIWI and MID domains was found to reduce the effective volume accessible to the target mRNA and therefore facilitate both the backbone coordination and base-pair formation. Further mutant simulations revealed that alanine mutation of the D358 residue on Helix-7 enhanced a trap state to slow down the loading of target mRNA. Similar trap state was also observed when wobble pairs were introduced in g6 and g7, indicating the role of Helix-7 in suppressing non-canonical base-paring. Our study pointed to a general mechanism for mRNA recognition by eukaryotic Agos and demonstrated the promise of qMSM in investigating complex conformational changes of biomolecular systems.

## Abstract (web summary)

Lizhe Zhu et al. use a quasi Markov State Model built from extensive molecular dynamics simulations to elucidate the dynamics of RISC-mRNA interactions. Their results provide further insight on how a target nucleotide is positioned before recognition by Argonaute.

## Introduction

Small non-coding RNAs (miRNA, siRNA) are critical for post-transcriptional regulation of human gene expression^1–4^. These RNAs are loaded into the Argonaute (Ago) protein, forming the RNA-induced silencing complex (RISC) which recognizes and inhibits target messenger RNA (target mRNA) in a highly sequence specific manner^5–12^. RISC regulates over 50% of human genes^13,14^ and is involved in numerous normal physiological functions^15,16^ and disease progression including cancer^17,18^. Accordingly, elucidating the mechanism of the recognition of target mRNA by RISC is expected to inspire the development of next-generation RNA-based therapeutics for cancer and other human diseases^19–21^.

**Fig. 1:**
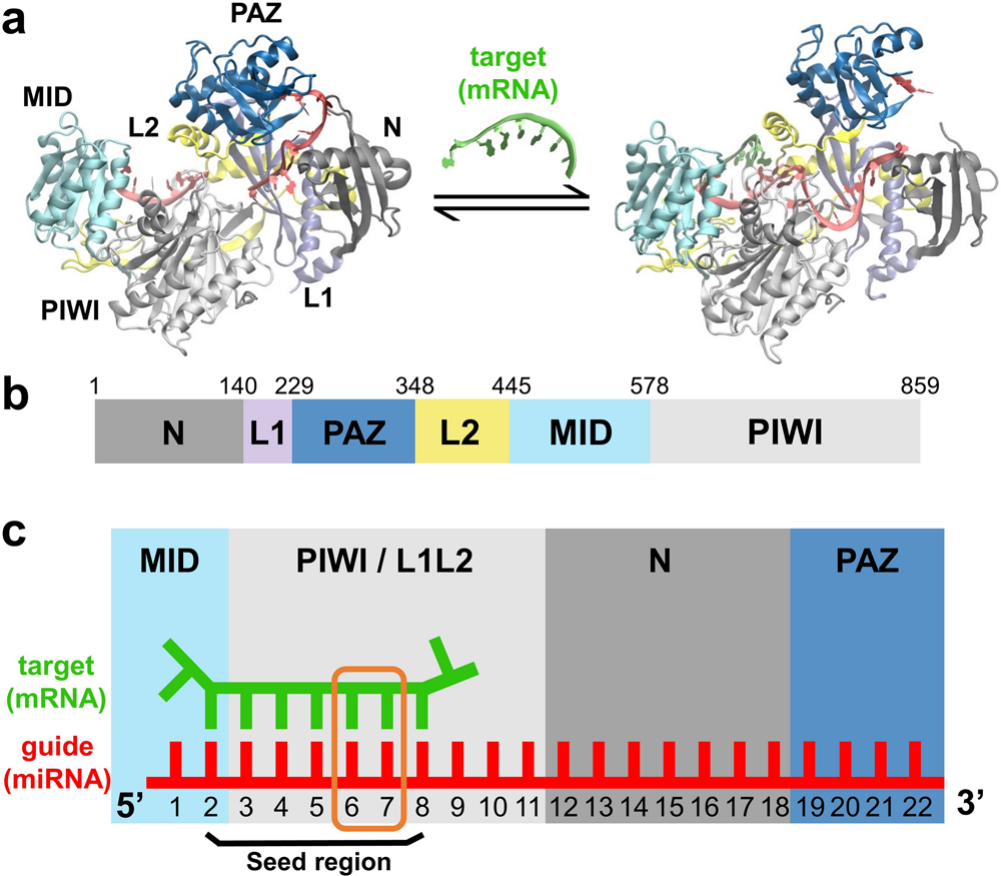
Function, domain decomposition and structure of the human Argonaute 2 (hAgo2). **a** Crystal structures of the RISC or hAgo2-miRNA complex (left, PDB ID: 4W5N) and RISC-mRNA complex (right, PDB ID: 4W5O). **b** Structural domains of hAgo2. **c** Cartoon illustration of target (mRNA) recognition by guide (miRNA) in hAgo2. The seed region (g2-g8) of guide is the labeled. Recognition at position g6-g7 studied in this work is highlighted in orange box.

Recent structural and single-molecule studies have pointed to a step-wise model^22–26^ of the recognition between target mRNA and the RISC complex formed by gRNA and the best studied human Argonaute human Ago 2 (hAgo2)^13,27–31^. The recognition initiates with the base-pairing process between the gRNA and target mRNA at position 2-8 (g2-g8) known as the seed region^14,27^ (Fig. 1). RISC first conducts a rapid diffusion-controlled search for the target mRNA sites that are complementary to the first half of the seed region (g2-g5). Subsequently, base pairs are formed at the second half of the seed region (g6-g8), accompanied by substantial conformational changes of hAgo2 that fully expose the gRNA g6-g8 for recognition. Though for certain gRNA sequences, base-pairing at g13-g16, known as the 3’ supplementary site, is also necessary, the seed base-pairing remains the most decisive step for the successful recognition and the translational repression of the target mRNA.

Existing crystal structures of the human RISC show that gRNA bases at g2-g6 are fully solvent-exposed and directly accessible to the mRNA while g7-g8 are much more buried by the PAZ and L2 domains of hAgo2^28^. In particular, Helix-7 (residues L356-T368 of L2 in hAgo2, conserved in eukaryotic Agos^32^) from L2 has direct contact with the mRNA at g6-g7 and causes a kink in the corresponding region in the gRNA-mRNA duplex^28,33^. This suggests that concerted motions of the PAZ and L2 are required to expose g7-g8 for the mRNA recognition, as validated in part by a recent biochemical and crystallographic effort^33^. However, due to the challenges for experimental techniques to resolve dynamics at the atomic level, fine details of the conformational dynamics of the RISC-mRNA complex, particularly hAgo2, during the seed recognition at g6-g7 have not been explicitly elucidated. Accordingly, the key protein residues that modulate the recognition process are also obscure.

Molecular dynamics (MD) simulations offer a valuable tool to investigate the conformational dynamics of large biomolecules at the atomic resolution. Previous MD studies at sub-microsecond timescales have demonstrated the impact of miRNA and double strand RNA on the conformational stability of the Ago complex^34–38^. However, solely using MD to study the target mRNA recognition at g6-g7 faces tremendous challenges due to the gap between the experimental timescale (at millisecond or longer) and that of MD simulations (at microsecond). The Markov state model (MSM) has been a popular framework to bridge this timescale gap^39–52^. In an MSM, we coarse grain both time and the conformational space into the lag-time Δt and a number of metastable states simultaneously, such that fast motions are integrated out. When Δt is longer than the intra-state relaxation time, the model becomes Markovian, i.e. the probability for the system to visit a conformational state at the next time step (t + Δt) is only determined by its location at the current time step t. If the model is Markovian, we can model the long timescale dynamics using the first order master equation. In recent years, MSM has been widely applied to study conformational dynamics of molecular recognition^53–59^ and aggregation^60,61^, including the mechanism of guide strand loading into hAgo2^62^. Since the lag-time (Δt) in an MSM must be long enough to allow Markovian interstate transitions, the estimation of transition probabilities in MSMs of the slow RISC-mRNA recognition could still be limited by the upper bound in the affordable length of the MD simulations. To address this issue, we recently developed the quasi-MSM (qMSM) method based on the generalized master equation formalism, which encodes non-Markovian dynamics into memory kernel functions^63^. qMSM has been applied to the study of bacterial RNA-polymerase^64^ and provides a promising approach to study the conformational changes during the RISC-mRNA recognition.

Therefore, we performed MD simulations that amounts to 84 microseconds and constructed a 4-state qMSM based on such a dataset. Our qMSM revealed that the slowest mode of motion of target mRNA recognition is not the gRNA-mRNA base-pairing, but the coordination between the phosphate groups of the mRNA and the positively charged residues of hAgo2 (R554, K550, K525, K355). Moreover, the positioning of Helix-7 is essential in facilitating this recognition: when Helix-7 approaches PIWI and MID, the effective 3D-space that needs to be explored by the target mRNA is reduced, therefore facilitating the backbone coordination and base-pairing. Further mutant simulations via metadynamics revealed that Alanine mutation of the negatively charged D358 on Helix-7 induced a conformational state that traps the system prior to the recognition at g7, which decelerated mRNA loading. A similar trap state was also observed when UG wobble-pairs are introduced at g6-g7. Altogether, our results suggest a backbone-coordination dominant and Helix-7 assisting mechanism for the target recognition by hAgo2, and thus highlight the vital role of protein-RNA interactions in this important biological process.

## Results and discussion

### qMSM reveals four conformational states during the target mRNA recognition

**Fig. 2:**
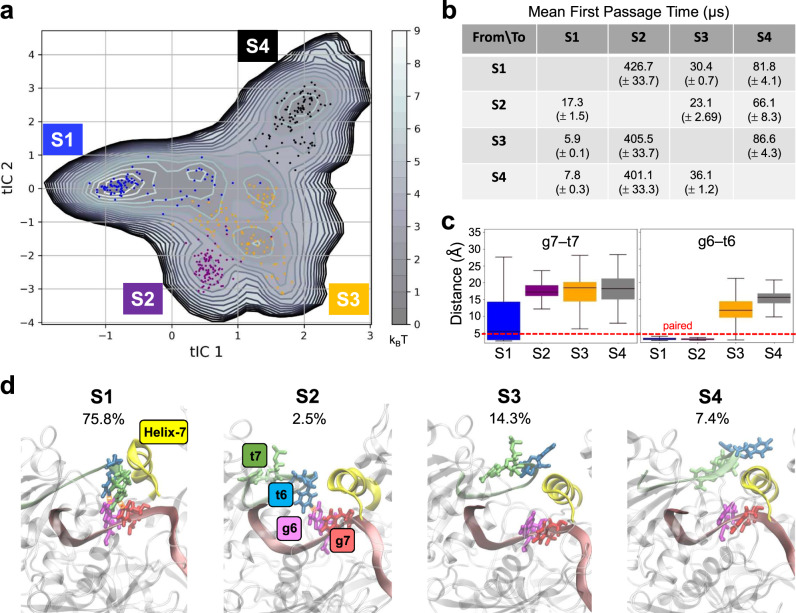
Markov State Models of target mRNA recognition at g6-g7. **a** the free energy landscape of the first two tICs. Samples from four macrostates (S1–S4) are labeled by blue, purple, orange and black, respectively. **b** The mean-first-passage-time (MFPTs) among the four macro-states. **c** Boxplot of the nucleobase distances at g6 and g7 for the four macrostates. Statistics were made on all structures in each macrostate. **d** Representative structure and the population of each macrostate. The MFPTs and population of the four macrostates are obtained from our qMSM.

Our qMSM contains four macrostates S1-S4. In Fig. 2a, we present the free energy landscape as a function of the first two time-lagged independent components (tICs). Obtained by the tICA analysis^65,66^, the tICs approximate the slowest modes of motions in our simulation data (see Methods). Macrostate S1, with a population of 75.8%, mainly corresponds to the recognized state where both base-pairs of g6-t6 and g7-t7 are formed (see statistics of the base distances in Fig. 2c) and Helix-7 moves away from MID and PIWI to accommodate the nucleotide t7 (first panel of Fig. 2d). Macrostate S3 and S4 denote two different unrecognized state (both base pairs broken, see Fig. 2c) with a population of 14.3 and 7.4% respectively. In State S3, the target mRNA stays close to MID (third panel in Fig. 2d) and the sidechain of t7 faces the solvent. In S4, the target mRNA locates far away from MID with a more extended conformation pointing to the hAgo2 protein (fourth panel in Fig. 2d). Macrostate S2, populated at 2.5%, corresponds to a metastable state where g6-t6 is formed while g7-t7 is not (see Fig. 2c and second panel in Fig. 2d).

Kinetically, the mean-first-passage-times (MFPTs) for the transitions to the mostly populated state S1 from other states (corresponding to the recognition process) are on timescales at several to tens of microseconds (see the first column of Fig. 2b), with significantly slower reverse transitions (see the first row of Fig. 2b). Meanwhile, the transitions to S2 from other states are all at ~400 μs (see the second column of Fig. 2b), significantly slower than the reverse transition (see the second row of Fig. 2b). This is consistent with the least population of S2 among all states and indicates that S2, though with t6 recognized, is kinetically less accessible from all other states. By contrast, the unrecognized states S3 and S4 are not only more populated than S2 but also kinetically close to S1 and to themselves. The recognition processes S3-to-S1 and S4-to-S1 only take 5.9 μs and 7.8 μs respectively, 2–3 times faster than the S2-to-S1 transition. Meanwhile, the interconversion between S3 and S4 occur on the same timescales (tens of microseconds) as the transition from S1 to them. These results indicate that the recognition from S3/S4 to S1 takes takes several microseconds and the t6-recognized and the least populated state S2 is an off-pathway intermediate state.

### Backbone phosphate coordination is rate-limiting for target recognition

**Fig. 3:**
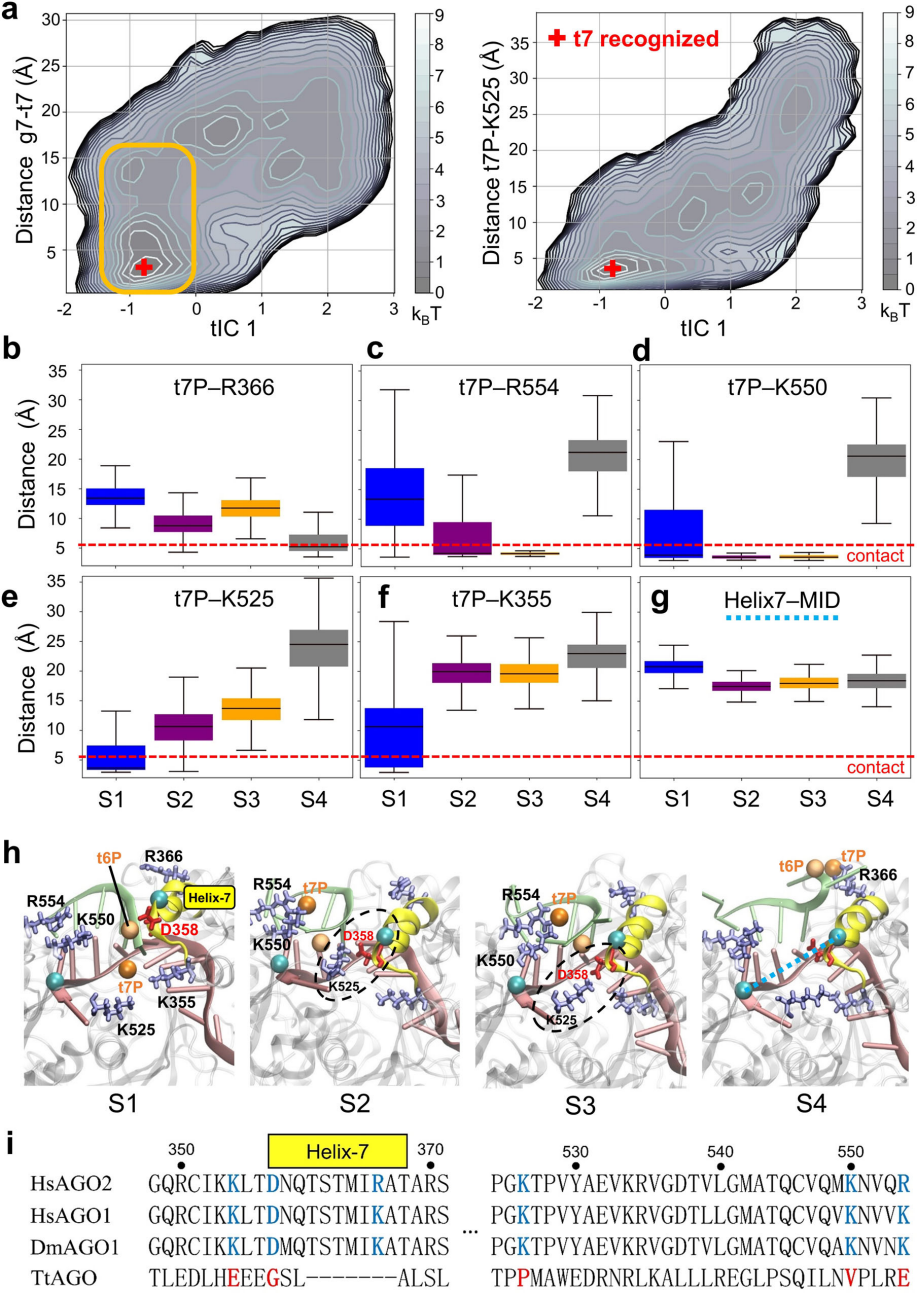
Key protein-RNA interactions during target recognition revealed by qMSMs. **a** Projection of the MSM data on the first tIC and the distances between the base of g7 and t7 (left), and between the phosphate of t7 (t7P) and the protein residue K525 (right). Orange box highlights the orthogonal distribution of the g7-t7 distance with respect to tIC1 near the fully recognized state. **b**–**g** Boxplot of various geometric features for the macrostates, including the distance between t7P and positively charged residues R366 / R554 / K550 / K525 / K355 and the distance between Helix-7 and MID domain (cyan dashed line in **c**). Atoms used for defining the distances are listed in SI Table S1. Statistics were made on all structures in each macrostate. **h** Representative structures of the four macro-states. The backbone phosphate t7P (orange sphere), the positively charged residues (ice-blue), Helix-7 (yellow), particularly the negatively charged D358 (red) on Helix-7 are highlighted. Cyan spheres in the S4 structure (right) are the Cα atoms of N359 and Q527 used to define the Helix-7-MID distance. Black dashed ellipses highlight the contact between D358 and K525 in S2/S3. **i** Sequence alignment for hAgo2, hAgo1, DmAgo1 and TtAgo. K355 / D358 / R366 /K525 / K550 / R554 are conserved in eukaryotic Agos.

To identify the slowest mode(s) of motions during target mRNA recognition, we examined the details of the RNA-RNA and protein-RNA interactions therein. As the first tIC from the tICA analysis approximates the direction of the slowest motion in our model, we projected the qMSM data on the first tIC (tIC1) and various geometric measurements. As shown in Fig. 3a, no clear correlation can be found between the tIC1 and the nucleobase distance g7-t7, since the distribution of this distance is nearly orthogonal to that of tIC1 (see the orange box in Fig. 3a) at the regions close to the recognized state (tIC1~−0.8). Instead, the change of the distance between the t7 phosphate (t7P) and the positively charged residue K525 exhibits notable correlation with tIC1 (right panel of Fig. 3a). These results suggest that it is the coordination between the nucleotide backbone phosphate with the positively charged protein residues that represents the rate-limiting step for the target mRNA recognition, rather than the formation of the base pairs at g6 and g7.

To further verify this observation, we made statistics on all MD conformations within each macrostate over the distances between t7P and all positively charged hAgo2 residues near the RNA loading channel (R366 / R554 / K550 / K525 / K355, Fig. 3b–f, definition in Table S1). Different macrostates have different phosphate-protein interactions. In macrostate S4, t7P is only in contact with R366 (Fig. 3b and fourth panel of Fig. 3h), whereas in S3, t7P is coordinated with R554 and K550 (Fig. 3c, d and third panel of Fig. 3h). In macrostate S2, t7P also forms contact with R554/K550 (second panel of Fig. 3h) but the R554 contact appears weaker than in state S3 (wider distribution in Fig. 3c). In the recognized state S1 (first panel of Fig. 3h), t7P turns to coordinate with K525 (Fig. 3e) and sometimes with K355 (Fig. 3f). Altogether, these results suggest that the transfer of t7P from R554/K550 or R366 to K525 appears a decisive step for target recognition (the S3-to-S1 and S4-to-S1 transitions). In fact, visual inspection of a MD trajectory where near-complete recognition is observed (Movie S1) shows that during recognition, t7P coordinates with the positively charged residues in a sequential manner R554- > R550- > K525- > K355. Projection of this trajectory on the first two tICs can be found in Fig. S1a. Detailed order of events can be found in Table S2 and Fig. S2 (see SI Note 1 for more discussion). Interestingly, all of these identified residues are highly conserved in eukaryotic Agos (see sequence alignment of hAgo2, hAgo1, DmAgo1 and TtAgo in Fig. 3i).

### Helix-7 facilitates target nucleotide positioning before recognition

As crystallographic studies have documented Helix-7 to pose a steric barrier for target mRNA recognition beyond g5^28,33^, we measured the distance between Helix-7 and the MID domain to investigate the role of Helix-7 in the recognition process (see definition in dashed cyan line in the fourth panel of Fig. 3h and Table S1). As expected, Helix-7 is 21.5 Å away from MID in the recognized state S1, significantly more distant than the 17–18.5 Å in state S2–S4 (Fig. 3g). Interestingly, in states S2 and S3, the negatively charged residue D358 on Helix-7 is even able to form a salt-bridge with K525 to keep Helix-7 proximal to MID/PIWI, partially closing the RNA-loading channel before recognition. As D358 is also highly conserved in eukaryotic Agos^32^ (Fig. 3i), we anticipate that the proximity between Helix-7 and MID/PIWI may play a pre-requisite role for the target mRNA recognition.

Given the aforementioned importance of phosphate coordination, we further hypothesized that the proximity of Helix-7 (e.g. driven by the salt-bridge between D358 in Helix-7 and K525 of hAgo2) could reduce the effective conformational space for t6 and t7 to explore and therefore facilitating the t7P coordination with K525 and the base-pairing. To examine this hypothesis, we designed two protein mutants K525A and D358A that disrupt the K525-D358 salt-bridge. In particular, we performed 1μs PCV-MetaD simulation for each mutant system to examine their impact on the target mRNA recognition. We identified one MD trajectory, in which a near-complete loading process is observed (see Fig. S1a and Movie S1) and extracted 36 structures from this MD trajectory to form a reference path. We then defined a PCV on this path and performed 1μs PCV-MetaD biasing on PCV-s and PCV-z for the wild-type (WT) protein and the two mutants (see Methods for details). In all the MetaD, the recognized state was revisited after complete unloading at least twice (Fig. S3), indicating sufficient sampling.

**Fig. 4:**
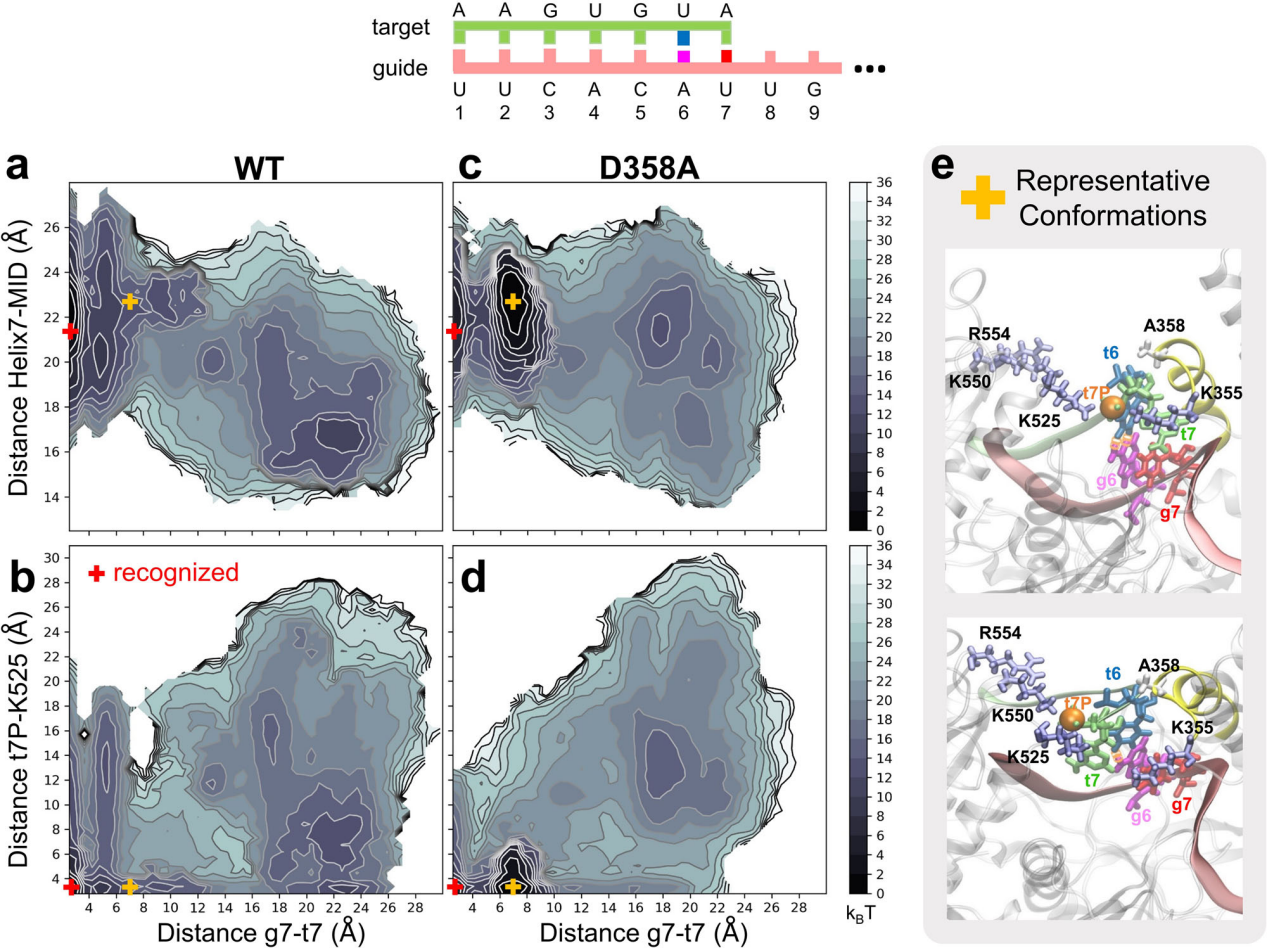
Metadynamics simulations of the wild type protein mutant D358A. **a**–**d** Reweighted free energy landscape of the g7-t7 distance, the distance between MID and Helix-7, and the distance between t7-phosphate and K525 are presented by contour plots. **e** Structures on the right are representative conformations for the D358A mutant extracted from the orange cross on the contour plots. The t7-phosphate (orange sphere), the positively charged residues (ice-blue), Helix-7 (yellow) and the A358 (white) are highlighted.

In Fig. 4a–d, we illustrate, for WT and the D358A mutant, the reweighted free energy landscape from the PCV-MetaD simulations as functions of three distances: (i) the Helix7-MID distance (*y*-axis of Fig. 4a, c) measuring the proximity of Helix-7 to MID/PIWI; (ii) the t7P-K525 distance (*y*-axis of Fig. 4b, d) that is statistically correlated to tIC1 (right panel of Fig. 3a); (iii) the g7-t7 base distance (x-axis in Fig. 4a–d) measuring the progress of t7-recognition (fully unrecognized >10 Å, in the recognition process 4.5-10 Å, fully recognized 3-4.5 Å). The results of WT PCV-MetaD simulations are qualitatively consistent with our qMSM data (Fig. S4).

For D358A, as expected, Helix-7 is distant (21–22 Å) from MID throughout the recognition process (Fig. 4c). As a result, a new trap metastable state that does not exist for WT emerged in D358A (see the orange cross in Fig. 4a–d and their conformations in Fig. 4e) right before the full recognition (g7-t7 distance at 7 Å) and therefore slowed down the recognition for D358A. We note that this new trap state is structurally distinct from any of the macrostates S1-S4 from qMSM of the WT system. In this trap state, Helix-7 is 23 Å away from MID (Fig. 4c), the t7P-K525 contact is formed (*y*-axis of Fig. 4d), and base-paring at g6 is formed (Fig. 4e). Similar trap state was also observed for the other mutant of K525A that disrupts the K525-D358 salt-bridge, due to the discouraged proximity of Helix-7 to MID/PIWI (Fig. S5). But the trap state in this mutant is less populated because A525 is no longer able to form stable contact with t7P.

Altogether, the above observations suggest a space-controlling role of Helix-7 in facilitating target mRNA recognition, rather than a previous hypothesis of Helix-7 pre-organizing the guide strand in the seed region^33^. Removal of negative charge on D358 widens the entrance of the RNA-loading channel, creates a trap state where only t6 is recognized, and consequently slows down the overall recognition.

### Helix-7 decelerates wobble pairing at g6-g7

Why is it necessary for Helix-7 to assist the target mRNA recognition in hAgo2? A previous FRET study has found that the facilitating role of Helix-7 was reduced if wobble pairs are introduced, i.e. the presence of Helix-7 discourages off-target recognition^33^. Therefore, we performed additional PCV-MetaD on WT and the D358A mutant with two wobble pairs at g6/g7 (wb67). We chose UG wobble pairs since it is known that the pairing free energy for a UG pair and a canonical pair are highly similar; the only difference is that a UG pair is ~1 Å longer than a canonical pair^67^, requiring larger space for accommodation. This introduced minimal alteration to the energetics and helped dissect the entropic effect of Helix-7 on the wobble pairs.

**Fig. 5:**
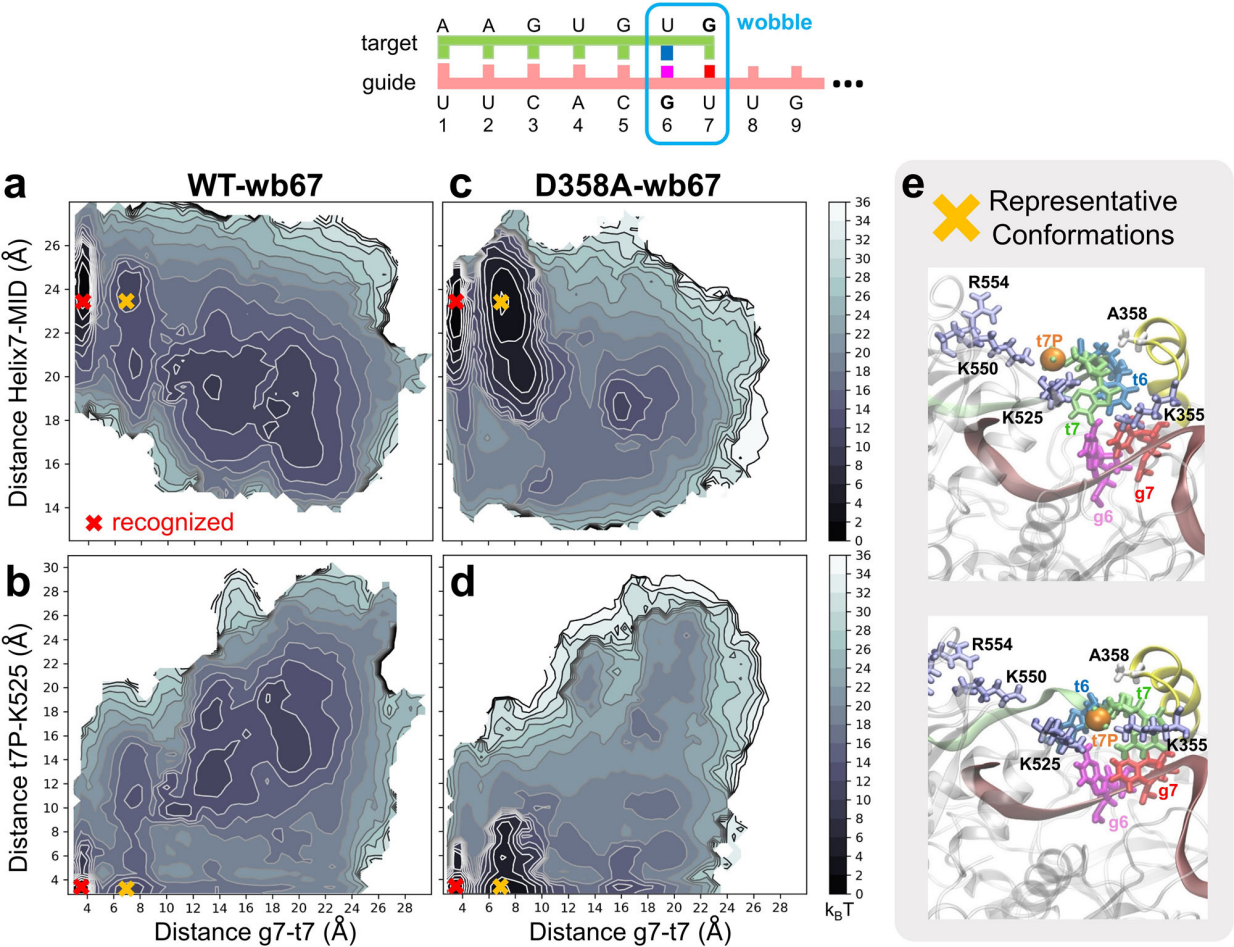
Metadynamics simulations of the wild type and mutant D358A protein with two UG wobble pair at g6 and g7. **a**–**d** Reweighted free energy landscape of the g7-t7 distance, the distance between MID and Helix-7, and the distance between t7-phosphate and K525 are presented by contour plots. **e** Structures on the right are representative conformations for the D358A mutant extracted from the orange cross on the contour plots. The t7-phosphate (orange sphere), the positively charged residues (ice-blue), Helix-7 (yellow) and the A358 (white) are highlighted.

As shown in Fig. 5a, b, the results for the WT-wb67 system are considerably different from WT (Fig. 4a, b), but similar to that of D358A (Fig. 4c, d). Not only Helix-7 has to be at least 24 Å away from MID to accommodate two wobble pairs (*y*-axis of Fig. 5a), a trap state (orange cross in Fig. 5a–d, representative conformations in Fig. 5e) similar to that of D358A (Fig. 4e) also emerges, indicating that the recognition of wobble pairs for the WT hAgo2 is in indeed slower than the canonical pairs, consistent with previous experimental results^33^. For D358A-wb67 (Fig. 5c, d), this trap state is similarly populated to the D358A system (Fig. 4c, d), despite the lacking of base-paring at g6 in the wobble pairs (Fig. 5e). These results suggest that Helix-7 can induce an off-pathway trap state when the wobble base pairs are present, and help hAgo2 to prevent the off-target recognition. Our observations provide a reasonable explanation for previous experimental findings^33^.

Argonaute is known to accelerate the formation of duplex between the gRNA and target mRNA through protein-RNA interactions^68^. Here we showed that backbone coordination of its target mRNA with the positively charged residues (K355 / R366 / K525 / K550 / K554) on hAgo2 is rate-limiting for the target mRNA recognition. The conservation of these residues in eukaryotic Argonautes (Fig. 3i) indicates a common recognition mechanism for them. Yet such backbone-coordination alone is not sequence dependent and thus cannot distinguish canonical base-pairs from uncanonical ones. By utilizing Helix-7, particularly the negatively charged D358 (also highly conserved in eukaryotic Argonautes only, see Fig. 3i), eukaryotic Argonautes, unlike their prokaryotic counterparts, developed an additional mechanism for fidelity control, i.e. regulating space at the entrance to the RNA-loading channel for the mRNA such that only the canonical base-pairing but not wobble-pairing is facilitated.

## Conclusion

We have constructed qMSM based on an extensive MD simulation dataset (84 μs in total) to elucidate the mechanism of target mRNA recognition by the hAgo2-gRNA complex at the second half of the seed region (g6-g7). Our qMSM revealed that the slowest mode for target mRNA recognition is not the gRNA- mRNA base-pairing, but the mRNA backbone phosphate coordination with the positively charged residues of hAgo2 (R366, R554, K550, K525, K355). Moreover, the positioning of Helix-7 (conserved in eukaryotic Agos) facilitates the recognition through controlling the effective conformation space to be explored by target mRNA. Alanine mutation of the negatively charged D358 on Helix-7 and UG wobble-pair mutants at g6-g7 both created a trap state prior to recognition at g7 and therefore decelerated the overall recognition. These results clearly illustrated the vital role of protein-RNA interaction in target mRNA recognition by eukaryotic Agos.

## Methods

### Structural modeling and molecular dynamics simulations

We built the structural models of hAgo2-gRNA-mRNA complex based on a crystal structure (PDB id: 4W5O) containing 9 base pairs (g2-g9)^28^. Homology modeling was performed to fill in missing part of the crystal structure via Modeller v.9.10^69^. We removed the nucleotides from both RNA strands at g8 and g9. This structure was solvated in a dodecahedron box with ~47,000 TIP3P waters, 144 Na+ and 143 Cl- ions, resulting in a total of ~156,000 atoms in the simulation box. The AMBER 99SB-IDLN force-field^70^ was used to describe the interactions in the system. Although the χOL3-correction of AMBER 99SB^71–73^ could have offered improved RNA backbone dynamics, we found that the χOL3-correction did not alter the transition state and therefore the recognition mechanism revealed in this work (see SI Note 2 and Fig. S6 for details). We used the software GROMACS v5.0.4 for MD simulations^74^. Energy minimization was performed for 10,000 steps by the steepest descent algorithm and then by the conjugate gradient algorithm. Then a 100 ps NVT simulations using the Berendsen thermostat^75^ was performed at 300 K for solvent equilibration, followed by a 1 ns NPT equilibration to 1 atm using the Berendsen barostat^75^. All MD simulations were performed in the NPT ensemble with a time-step of 2 fs, the v-rescale thermostat^76,77^ and the Parrinello-Rhamann barostat^78^. Long-range electrostatic interactions were treated by the Particle-Mesh Ewald method^79^. The short-range electrostatic and van der Waals interactions both used a cut-off of 10 Å. All bonds were constrained by the LINCS algorithm^80^.

### Initial sampling of base-pair disruption at g6-g7 via metadynamics simulations

Directly simulating the target mRNA recognition via conventional MD simulations is extremely challenging because hAgo2, miRNA and mRNA are all large and flexible biomolecules. We have applied metadynamics (MetaD) to obtain sufficient initial sampling^81,82^. Starting from the structural model of RISC-mRNA complex with base pairs formed at g2-g7, we performed MetaD simulations via PLUMED^83^, with bias potentials on the base distances at g6-g7 to sample the disruption and re-formation of the two base-pairs (details in SI Note 3). Four independent MetaD simulations were performed. In all four simulations, multiple rounds of breaking and re-formation of the two base-pairs were observed (Fig. S7), indicating sufficient initial sampling. Note that no breaking of the base-pairs or base-stacking at g2-g5 was observed during the metadynamics or subsequent unbiased simulations (see SI Note 4 and Fig. S8 for details).

### Microstate-MSM construction and validation

We then performed K-centers clustering on the four MetaD trajectories and obtained 84 clusters. Starting from the geometric center conformations of these 84 clusters, we performed unbiased MD simulations for MSM construction, resulting in 84 trajectories with each in length of 1 μs, with a saving interval of 200 ps. Based on this MD dataset, we followed our previously published protocol^49,84^ to construct microstate-MSM to investigate the RISC-mRNA recognition. Time-lagged Independent Component Analysis (tICA)^65,66^ was first used for dimensionality reduction. Spectral oASIS^85^ was used to reduce the number of input features (Fig. S10). The APLoD algorithm^86^ was then used to define microstates in the reduced tIC space. Throughout this procedure, we applied the Generalized Matrix Rayleigh Quotient (GMRQ)^87^ score to evaluate the quality of the model (Fig. S9c–e). The final microstate MSM was built on the first 4 tICs with 81 microstates (details in SI Note 5-7), which is validated by the Chapman-Kolmogorov test (Fig. S11b, details in SI Note 7). Construction of the microstate MSM was performed using our in-house python code based on MSMbuilder version 3.8.0^88^. The free energy landscapes for the microstate MSM data were visualized by MSMExplorer^89^.

### Macrostate *quasi*-MSM construction and validation

To assist the interpretation of target-recognition mechanisms, we applied our recently developed qMSM^63^ approach to construct a model containing 4 macrostates (state S1–S4). To obtain this qMSM, we first performed the kinetic lumping to group 81 microstates into 4 macrostates using the PCCA + algorithm^90,91^ implemented in PyEMMA version 2.5.2^92^, because a stable gap is observed between 3rd and 4th slowest implied timescale (details in SI Note 7, 8). Our qMSM applies the generalized master equation formalism to encode the non-Markovian dynamics in time-dependent memory kernels (${{{{{\boldsymbol{K}}}}}}\left(\tau \right)$):

1 $$\dot{{{{{{\boldsymbol{T}}}}}}}\left(t\right)={{{{{\boldsymbol{T}}}}}}\left(t\right)\dot{{{{{{\boldsymbol{T}}}}}}}\left(0\right)+{\int }_{0}^{{{\min }}\left\{t,{\tau }_{K}\right\}}{{{{{\boldsymbol{T}}}}}}\left(t-\tau \right){{{{{\boldsymbol{K}}}}}}\left(\tau \right)d\tau ,$$

where ${\tau }_{K}$ corresponds to the memory kernel relaxation time where ${{{{{\boldsymbol{K}}}}}}\left(t \, > \, {\tau }_{K}\right)\approx {{{{{\bf{0}}}}}}$, and ${{{{{\boldsymbol{T}}}}}}\left(t\right)$ refers to the TPM. We validated We validated our final qMSM via the Chapman-Kolmogorov test and compute MFPTs among the four macrostates via the transition path theory^93,94^ (details in SI Note 8, 9).

### Setup of the Path-Collective-Variable metadynamics simulations for mutants

To verify the predictions from the qMSM, we designed several protein mutants and a RNA mutant with two UG-wobble-pairs at position 6 and 7. To perform conformational sampling of these mutant systems, we conducted 1 μs long well-tempered^82,95^ Path-Collective-Variable (PCV) MetaD simulations^96^ for each mutant as well as the wild-type. PCV-MetaD is a MetaD simulation biasing on two PCV-s and PCV-z, denoting the progress along and the average distance from a high dimensional reference path respectively, given a pre-defined distance metric. Our reference path was extracted from one of the 84 MD simulation trajectories, in which a near-complete recognition process can be observed (Fig. S1a and Movie S1). The reference path consisted of 36 nodes with a RMSD of ~1.4 Å between neighbor nodes (28 nodes for the H7G5 mutant, where Helix-7 was replaced by 5 glycine residues). RMSD is measured by two atom-sets: (i) structural alignment was performed on the Cα atoms of the MID and PIWI domain; (ii) RMSD was calculated using the Cα of Helix-7, all heavy atoms from target nucleotides t6/t7 and the sidechains of R554, K550, Q548, K525, K355. The widths of Gaussian hills for PCV-s and PCV-z were chosen as 0.5 and 3Å^2^ respectively. These Gaussian hills of height 1.25 kJ/mol were deposited every 200 ps with a bias-factor of 15 at reference temperature of 310 K. To ensure efficient sampling, we imposed a wall potential at PCV-z = 36 Å^2^, below which the majority of MSM samples were encompassed (Fig. S1b). Analysis of the free energy landscapes over the collective variables (CVs, with physical meanings) other than s and z were obtained via a standard reweighting procedure^97^. To ensure the smoothness of the reweighted landscape, values of the CVs were recorded every 10 ps.

### Statistics and reproducibility

Though MD simulations at constant temperature are stochastic in nature, the statistics are reproducible if sufficient sampling, under the framework of MSM and qMSM as in this manuscript, is achieved. We performed homology modeling via the software MODELLER (https://salilab.org/modeller/). MD simulations were performed via GROMACS version 5.0.4 (http://www.gromacs.org). The mutant metadynamics simulations were performed via the PLUMED plugin (https://www.plumed.org). The MSM and qMSM were built via the MSMbuilder version 3.8.0 (http://msmbuilder.org) and PyEMMA version 2.5.2 (http://emma-project.org/latest/).

### Reporting summary

Further information on research design is available in the Nature Research Reporting Summary linked to this article.

## Supplementary information

Supplementary Information

Description of Additional Supplementary Files

Supplementing Movie 1

Supplementary Data 1

Reporting Summary

## Data Availability

Data underlying main figures is presented in Supplementary Data 1. The authors declare that all other data supporting the findings of this study are available within the paper and its supplementary information files, or are available from the corresponding author upon reasonable request.

## Code availability

All in-house code is available from the corresponding authors upon reasonable request.
