# Supplementary material for: Critical role of backbone coordination in the mRNA recognition by RNA induced silencing complex
Source: Commun Biol. 2021 Nov 30;4:1345. doi: 10.1038/s42003-021-02822-7 (PMC8632932; doi:10.1038/s42003-021-02822-7)
Supplement: Supplementary file 1 — Supplementary Information [file 42003_2021_2822_MOESM1_ESM.pdf]

Supplementary Information

Critical Role of Backbone Coordination in the mRNA recognition by RNA Induced Silencing Complex

Lizhe Zhu[1,2], Hanlun Jiang[3,4], Siqin Cao[2,5], Ilona Christy Unarta[3,5], Xin Gao[6]* and Xuhui Huang[2,3,5]*

[1] Warshel Institute for Computational Biology, School of Life and Health Sciences, The Chinese University of Hong Kong (Shenzhen), Shenzhen, Guangdong 518172, China
[2] Department of Chemistry, The Hong Kong University of Science and Technology, Clear Water Bay, Kowloon, Hong Kong
[3] Department of Chemical and Biological Engineering, The Hong Kong University of Science and Technology, Clear Water Bay, Kowloon, Hong Kong
[4] Department of Biochemistry, Institute for Protein Design, University of Washington, Seattle WA 98195, United States of America
[5] Center of Systems Biology and Human Health, State Key Laboratory of Molecular Neuroscience, The Hong Kong University of Science and Technology, Clear Water Bay, Kowloon, Hong Kong
[6] Computational Bioscience Research Center, Computer, Electrical and Mathematical Sciences and Engineering Division, King Abdullah University of Science and Technology (KAUST), Thuwal, 23955-6900, Saudi Arabia

* To whom correspondence should be addressed. Tel: (852) 23587363; Email: xuhuihuang@ust.hk; Correspondence may also be addressed to xin.gao@kaust.edu.sa

Supplementary Note 1. Transition Path Theory (TPT) analysis reveals detailed order of event for the target mRNA recognition

To elucidate a more detailed sequence of events, we also performed the TPT analysis based on the microstate-MSM (containing 81 microstates) to find the highest flux paths connecting representative microstate(s) of the macrostate S4 with those in macrostate S1. For macrostate S4 (i.e. the initial state), we selected microstate 43, which is at the geometric center of S4. For macrostate S1 (i.e. the final state), we selected microstate 5 and 26 corresponding to the crystal structure of the recognized state. Our TPT analysis reveals that the top 10 highest flux paths cumulatively account for 48.6% of the overall flux of top 100 high-flux paths (see Table S2).

We show in Fig.S2 the representative conformations of top 3 highest-flux paths obtained from the microstate MSM, and these 3 paths amount to 27.64% of the total flux. The order of events is as follows:

(a) To leave macrostate S4, the phosphate of t7 (t7P) has to be detached from R366. This leads to a state where t7P forms no contact with any positively charged residue (microstate 55, cyan in Table R1 or microstate 57, red in Table R1).

(b) Then t7P may choose to coordinate with either K550/R554 (microstate 36) together or K550 alone (microstate 67) with the t6/t7 nucleobases sit at the entrance of the RNA-loading channel.

(c) Finally, as Helix-7 moves away from the entrance and t7P coordinates with K525, the t6/t7 nucleobases manage to enter the RNA-loading channel and form base-pairs with the guide RNA at g6/g7.

Supplementary Note 2. Comparison of different RNA force field parameters

To explore the potential influence of the RNA force field parameters on the found mechanism, we selected a conformation from the most frequently occurred microstate in the top 10 highest-flux paths between S1 and S4 (bold in Table S2). This conformation (Fig.S6a) is located close to the transition state region between S1 and S4, as illustrated by its committor analysis (Fig.S6b). For the committor analysis, we performed ten 2ns-MD simulations starting from this conformation with different random initial velocities. The projection of these 10 trajectories onto the first two tICs showed that 5 trajectories relax towards the macrostate S4 (black lines in Fig.S6b) and 5 trajectories relax towards macrostate S1 (blue lines in Fig.S6b), indicating that this conformation is located near the transition state region between S1 and S4.

To further examine the potential impact of the RNA force field, we performed 10 extra 2ns-MD simulations from the same conformation using the improved force field with the χOL3 corrections[1]. Fig.S6c shows that the range of sampling in the space of the first two tICs for the improved force field (orange) is similar to ff99SB (black). These results indicate that the transition state region is not altered when the force field parameters were changed from AMBER 99SB-IDLN to the χOL3-corrected version. We anticipate that this could be due to the fact that the χOL3-correction aimed at discouraging the ladder-like artifact of AMBER 99SB-IDLN that occur only when the nucleobases are closely packed[1-3], while in the transition state region, the bases of t6, t7 are distant from those of g6 and g7.

Supplementary Note 3. Initial metadynamics sampling

The construction of Markov State Models relies on large collection of unbiased MD trajectories, whose seeding structure must first be provided, requiring an extensive initial sampling before the large-scale shooting simulations. To obtain the seeding conformations covering the recognition process, we performed four independent metadynamics (MetaD)[4, 5]. Starting from the structural model of RISC-mRNA complex with base pairs formed at g2-g7, we performed two dimensional MetaD simulations via the PLUMED plugin[6], with bias potentials on the base distances at g6-g7 to sample the disruption and

re-formation of the two base-pairs. The base distances were measured via the central Nitrogen atom of the base group. The Gaussian hills of width 0.5Å and height 2.5kJ/mol was deposited every 10ps. In all four simulations (each of length 200ns), multiple rounds of breaking and re-formation of the two base-pairs have been observed (Fig.S7).

Supplementary Note 4. Stability of base-pairs and base-stacking at the first half of the seed region

Since the sequential recognition model for hAgo2 has been well established in the literature[7-9], we followed this model to assume that the stability of the base-pairs at g2-g5 is a pre-condition for subsequent recognition at g6-g8. Accordingly, no bias potential was added to disrupt the base-pairs at g2-g5 during the initial four metadynamics simulations. These base-pairs remain stable also throughout all subsequent MD simulations used to build the microstate MSM and macrostate qMSM. Such stable base-pairing also helps stabilize the stacking among the consecutive nucleobases in both the guide and target RNA strand at g2-g5.

To demonstrate this, we computed the center of mass (c.o.m.) distance between the consecutive nucleobases in both strands at g2-g5 using all our MSM data. Fig.S8 shows the corresponding boxplot of these base distances. The narrow distributions of all these base c.o.m. distances indicate that no breaking of the base-stacking occurred at g2-g5 throughout our MD simulations.

Supplementary Note 5 Selecting features as input for tICA

We performed dimensionality reduction of the all-atom MD conformations using time-lagged Independent Component Analysis (tICA)[10, 11]. To select input features for tICA, we first identified all the protein and RNA residues that are in contact with target mRNA at g6 and g7 for at least 10% of the simulation frames in each Metadynamics simulation (initial sampling simulation) using g_contacts[12]. Finally, 20 protein residues, 6 gRNA residues, and 3 mRNA residues were selected. For these protein and RNA residues, we selected two atoms to represent each residue, i) the C-α atom and the C atom furthest from the backbone for protein, ii) P atom and N atom furthest from the backbone for RNA. This results in an atom set containing 57 atoms. We then used all-to-all pairwise distances between the atoms in the atom set (1540 features) as input for Spectral oASIS method[13] to further reduce the number of atom pairs. We calculated the slowest tICA implied timescale using two tICA lag time values, 250 ns and 300 ns (Fig.S9a-b). Based on Fig.S9, the covariance matrix built from one-thousand features selected by Spectral oASIS can well approximate the slowest tICA implied timescale of the full matrix with 1540 features.

Supplementary Note 6 Choice of hyperparameters for the tICA and microstate clustering

To find the optimal features, tICA hyperparameter (number of tICS and tICA lag time), and APLoD parameter, we employed variational cross-validation technique (Generalized Matrix Rayleigh Quotient, GMRQ)[14]. We used six-fold cross validation to calculate the GMRQ score of the train and test data set. We found that the 1000 features chosen by Spectral oASIS with tICA relaxation time of 300 ns produce the largest GMRQ score in the test set (Fig.S9c). The pairwise distances selected by Spectral oASIS are shown in Fig.S10. We also determined that 4 tICs and k-nearest neighbor of 250 for APLoD[15] clustering (81 microstates) are the optimal parameter based on the GMRQ score (Fig.S9d-e). For all the calculation of GMRQ scores, the MSM lag time was 380 ns. The tICA and GMRQ tests were performed using the in-house python code based on MSMbuilder version 3.8.0[16].

Supplementary Note 7 Validation of microstate-MSM and Lumping to macrostates

We construct an 81-microstate MSM at the lagtime of 380ns, as the implied time scale plot levels off at this lag time (Fig.S11a). We also validated the microstate-MSM using the Chapman-Kolmogorov test

and showed that the residence probabilities from the eight most populated microstates predicted by the microstate-MSM are consistent with those directly counted from all-atom MD simulations (Fig.S11b). The transition probability matrix for this microstate MSM is given in Fig.S12.

To enable the interpretation of biological mechanisms, we then performed kinetic lumping of the microstates (81 microstates) to four macrostates using the PCCA+ algorithm[17] implemented in PyEMMA version 2.5.2[18]. Four macrostates were chosen since a stable gap in the implied timescale plots occurs between the 3rd and 4th slowest timescale (Fig.S11a).

Supplementary Note 8. Macrostate quasi-MSM construction and validation

To model the long timescale dynamics of RISC-mRNA recognition, we further built quasi Markov State Model (qMSM)[19] for the four-state model. The four-state Markov State Model obtained by lumping is not Markovian within all available lag times in our MD trajectories, which can be seen in Fig.S13 where the 4-state MSM models built at lag times 50ns or 80ns deviated significantly from the MD simulations. Thus, we employed qMSM to study the dynamics of the four-state model. Our qMSM can correctly predict the time-dependent transitions ($\boldsymbol{T}(t)$) using the generalized master equation (Eq. (1) in the main text) and explicit consideration of memory kernels ($\boldsymbol{K}(t)$).

In our numerical implementation, we have employed the following form of the generalized master equation that discretised on a time sequence $t = n\Delta t$ ($\Delta t = 1\text{ns}$ in this study):

$$\dot{\boldsymbol{T}}(n\Delta t) = \boldsymbol{T}(n\Delta t)\dot{\boldsymbol{T}}(0) + \sum_{m=1}^{\min\{n,\tau_K/\Delta t\}} \boldsymbol{T}(n\Delta t - m\Delta t)\boldsymbol{K}(m\Delta t)\Delta t \quad \text{(S1)}$$

Therefore, the memory kernels on the discretized time sequence are calculated by the following equation:

$$\boldsymbol{K}(n\Delta t) = \frac{\dot{\boldsymbol{T}}(n\Delta t) - \boldsymbol{T}(n\Delta t)\dot{\boldsymbol{T}}(n\Delta t)}{\Delta t} - \sum_{m=1}^{n-1} \boldsymbol{T}(n\Delta t - m\Delta t)\boldsymbol{K}(m\Delta t) \quad \text{(S2)}$$

In this paper, the input TPMs from 1ns to 160ns (i.e. $T(1\text{ns}), T(2\text{ns}), \dots T(160\text{ns})$) were calculated by counting the transitions $C_{ij}(n\Delta t)$ from MD trajectories: $T_{ij}(n\Delta t) = \left(C_{ij}(n\Delta t) + C_{ji}(n\Delta t)\right) / \sum_j \left(C_{ij}(n\Delta t) + C_{ji}(n\Delta t)\right)$, where the detailed balance was enforced by direct symmetrization (transpose) [20, 21]. However, the time dependent TPMs that are directly counted from transitions have large numerical fluctuations. Thus, we adopted the following smoothing scheme to reduce numerical fluctuations:

$$\boldsymbol{T}_s(t) = \boldsymbol{V}(t)\boldsymbol{\lambda}_s(t)\boldsymbol{V}(t)^{-1} \quad \text{(S3)}$$

where $\boldsymbol{V}(t) = [V_1(t), V_2(t), V_3(t), V_4(t)]$ is a 4×4 matrix of the right eigenvectors ($V_i(t)$) of the original $\boldsymbol{T}(t)$ that is directly counted from MD trajectories, and $[\boldsymbol{\lambda}_s(t)] = \text{diag}\left\{\lambda_1^{(\text{s})}(t), \lambda_2^{(\text{s})}(t), \lambda_3^{(\text{s})}(t), \lambda_4^{(\text{s})}(t)\right\}$ is a diagonal matrix of smoothed eigenvalues. The smoothed eigenvalues are obtained by a triple-exponential fitting of the original TPMs:

$$\lambda_i^{(s)}(t) = a_{i1}e^{-b_{i1}t} + a_{i2}e^{-b_{i2}t} + a_{i3}e^{-b_{i3}t} + c_i \quad \text{(S4)}$$

where $(a_{i1}, a_{i2}, a_{i3}, b_{i1}, b_{i2}, b_{i3}, c_i)$ are the seven parameters to be fitted.

The memory kernels $\boldsymbol{K}(\tau)$ were computed at $t = [0, 1\text{ns}, 2\text{ns}, \dots, 159\text{ns}]$. The memory kernels lifetime τ_K can be obtained from the mean integration of memory kernel (MIK) which was defined as follows,

$$MIK(t) = \frac{1}{4}\sqrt{\sum_{i,j=1}^{4}\left(\int_0^t K_{ij}(t')dt'\right)^2} \quad \text{(S5)}$$

The MIK should reach a plateau at the memory kernel lifetime τ_K when memory kernels fully decay. In this paper, we found that the memory kernel lifetime is $\tau_K = 50\text{ns}$ (Fig.S13a). The qMSM with the

memory kernel lifetime $\tau_K = 50\text{ns}$ is validated by the Chapman-Kolmogorov test (Fig.S13c), where the qMSM with $\tau_K = 50\text{ns}$ can accurately predict the residence probabilities for all four metastable states directly counted by MD simulations. In a sharp contrast, the four-state MSM model at 80ns lag time (the longest lag time we can use to validate MSM in our MD trajectories) still have large deviations from the MD simulations. The deviation of predictions can be quantified by the root mean square errors (RMSEs) between the predicted dynamics and MD simulations, which is defined as follows in this paper:

$$\mathrm{RMSE}_x(\tau_x, L_x) = \sqrt{\frac{1}{16L_x}\sum_{n=1}^{L_x}\sum_{j,k=1}^{4}\left[T_{jk}^{x}(n\delta t_x) - T_{jk}^{MD}(n\delta t_x)\right]^2} \quad \text{(S6)}$$

Where x stands for algorithm (MSM or qMSM). $L_x = 160\text{ns}/\tau_x$. $\delta t_{MSM} = \tau_{MSM}$ and $\delta t_{qMSM} = \Delta t$. T^{MD} are directly counted from MD simulations.

To sum, we used the discretized generalized master equation as shown in SI Eq.S1-S2 to numerically compute $\boldsymbol{K}(t)$ at $t = n\Delta t$ (Δt=1ns)[19]. Here, we selected $\tau_K = 50\text{ns}$ as the mean integration of memory kernel (MIK) converges at this time (Fig.S13a and Eq.S5). We validated our qMSM using the Chapman-Kolmogorov test by showing that the qMSM can reproduce residence probabilities for all four states (red curves in Fig.S13c). In contrast, the 4-macrostate MSM without considering the memory kernels was not Markovian even with a lag time up to 80ns (green curves in Fig.S13b,d). In addition, these first-order MSMs predicted significantly faster dynamics than those of the MD simulations in the Chapman-Kolmogorov test (green curves in Fig.S13c,d), and also predicted substantially shorter mean first passage times (MFPTs) than qMSM (Fig.S14). To compute MFPTs among the four macrostates by the transition path theory[22, 23], we utilized the TPM: $T(\tau = 1\mu\text{s})$ obtained from the discretized generalized master equation in our qMSM.

Supplementary Note 9. Calculation of mean first passage time (MFPTs) between pairs of metastable states from qMSM

The MFPTs between states can be computed from the 4-state dynamics of qMSM. First the TPMs at different lag times were either obtained by the generalized master equation in our qMSM (when lag time is longer than 400ns, see Eq.S1) or counted from MD simulations (when lag time is no longer than 400ns). Then we performed MCMC simulations based on these TPMs, and MFPTs between each pair of states can be computed from MCMC trajectories. To estimate the standard deviation of MFPTs, we produced 20 independent MCMC simulations with 10^5 steps. The MFPTs between recognized state (S1) and unrecognized state (S4) as a function of lag time are shown in Fig.S9. The MFPTs of qMSM converges at lag time $(\tau) = 1\mu\text{s}$ (Fig.S14). Therefore, our reported MFPTs (main-text Fig. 2b) were calculated based on TPM obtained by qMSM at lag time=1μs. The 4-macrostate MSM predicts faster MFPTs than qMSM for both S1-to-S4 and S4-to-S1 transitions (Fig.S14b,d), as the dynamics of 4-state model are not Markovian at these lag times.

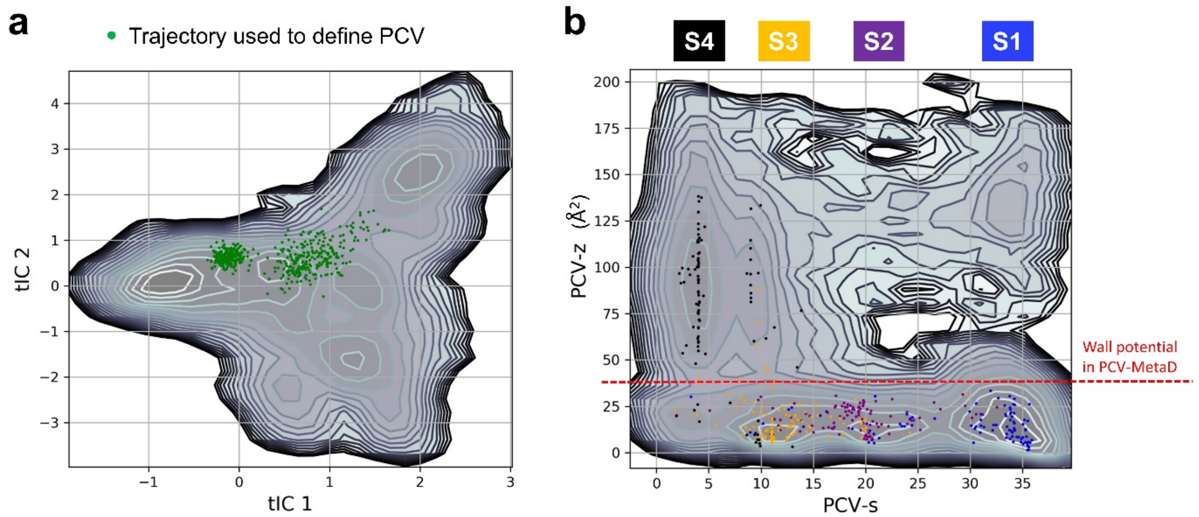

Supplementary Figure S1. (a) Projection of the extracted loading trajectory on the first two tICs. **(b)** Projection of the MSM data on the path-collective-variables defined on the extracted trajectory. Wall potential was introduced on PCV-z=36Å^2 covering the majority of highly populated regions.

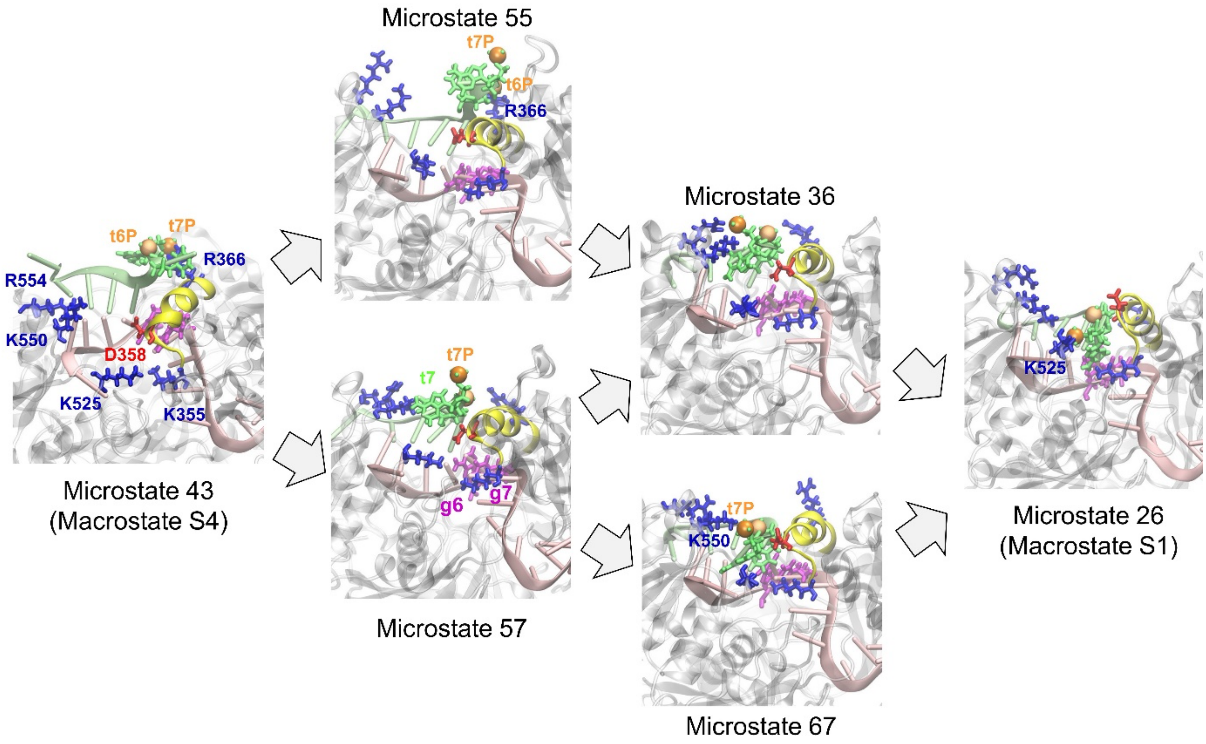

Supplementary Figure S2. Representative conformations of the S4->S1 transition illustrating the order of events during target mRNA recognition. The top 3 paths of the microstate MSM are visualized since they amount to 27.64% of the total flux (see Table S1). To leave macrostate S4, the phosphate of t7 (t7P) has to be detached from R366. This leads to a state where t7P forms no contact with any positively charged residue (microstate 55, cyan in Table S1 or microstate 57, red in Table S1). Then t7P may choose to coordinate with either K550/R554 (microstate 36) together or K550 alone (microstate 67) with the t6/t7 nucleobases sit at the entrance of the RNA-loading channel. Finally, as Helix-7 moves away from the entrance and t7P coordinates with K525, the t6/t7 nucleobases manage to enter the RNA-loading channel and form base-pairs with the guide RNA at g6/g7.

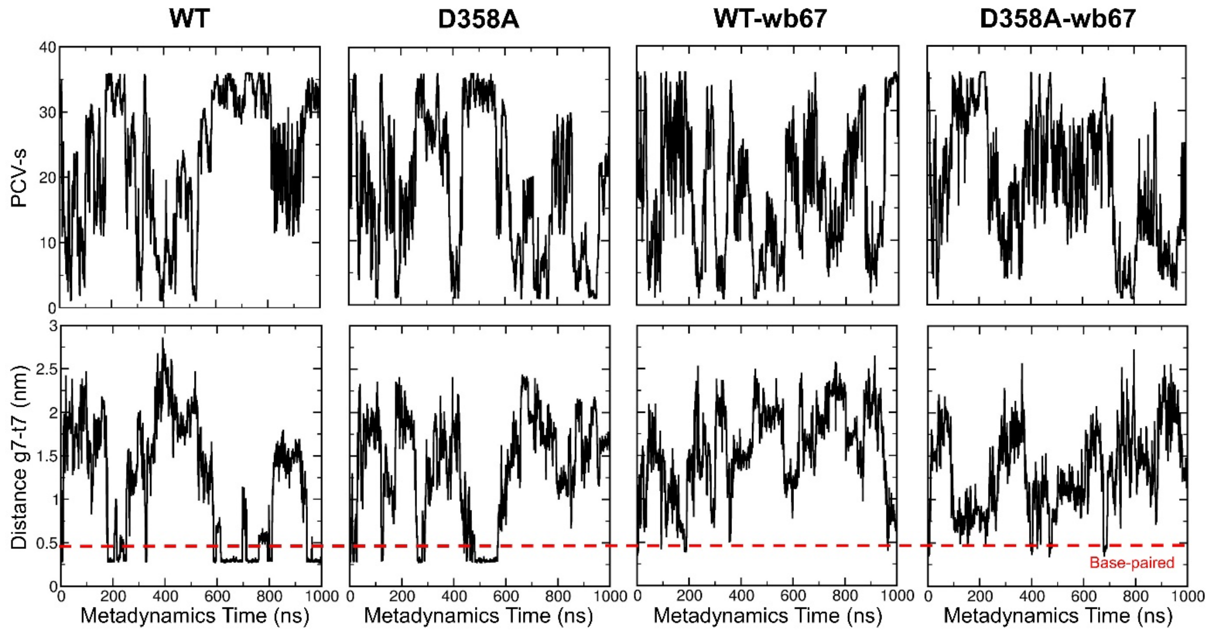

Supplementary Figure S3. Time-traces of PCV-s and the base distance of g7-t7 for all PCV-Metadynamics simulations of the four systems (wild-type and D358A protein with and without wobble pairs at g6 and g7). Multiple round trips of the PCV-s (between 1 and 36) and multiple re-formation of the g7-t7 base-pair indicate reasonably sufficient sampling.

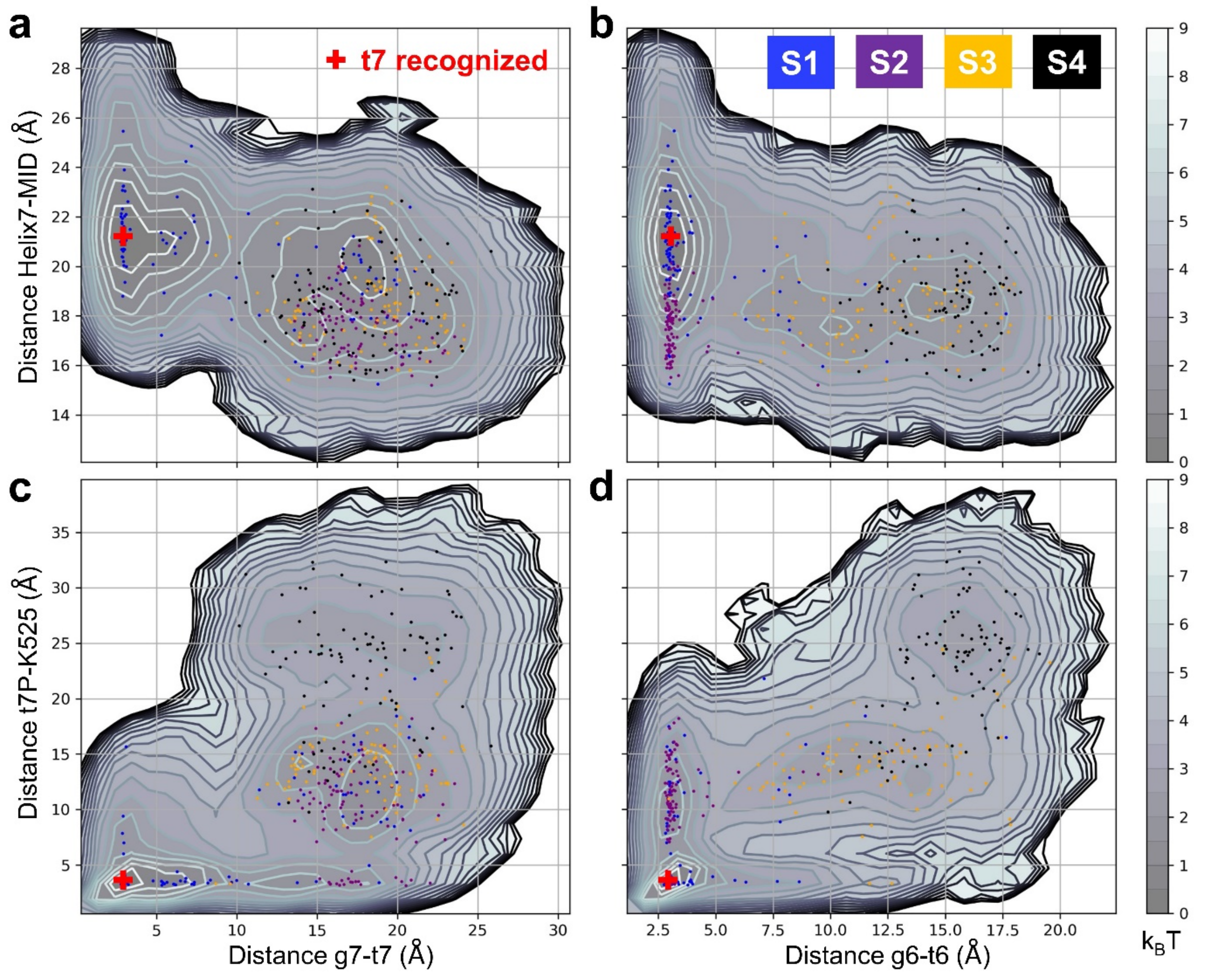

Supplementary Figure S4. Projection of qMSM data on four distances between g7 and t7 (panel a,c), between g6 and t6 (panel b,d), between Helix-7 and the MID domain (panel a,b), and between t7P and the protein residue K525 (panel c,d). The four macrostates S1-S4 are labelled blue, purple, orange and black respectively.

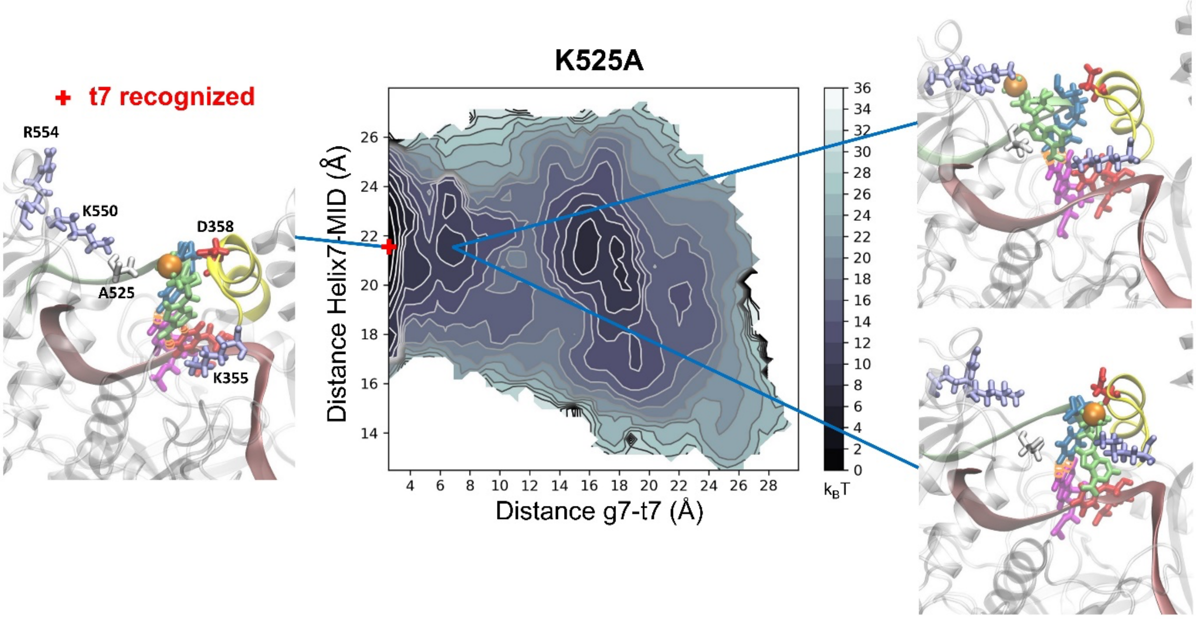

Supplementary Figure S5. Metadynamics simulations of the protein mutant K525A.

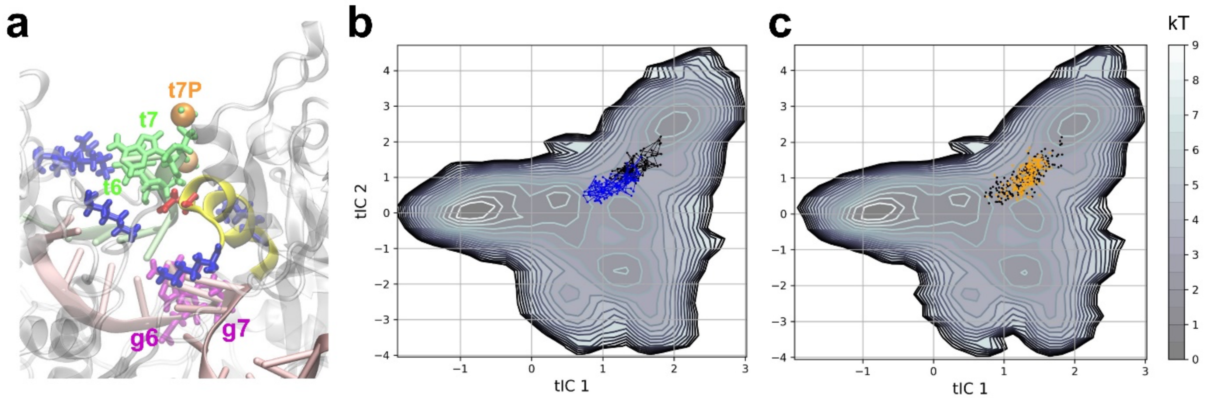

Supplementary Figure S6. Sampling of the transition state region for the S4->S1 transition. (a) the conformation from which sampling was initiated. **(b)** Projection of AMBER 99SB-IDLN trajectories on the first two tICs. Blue and black lines correspond to the 50% trajectories relaxing to macrostate S1 and 50% trajectories relaxing to macrostate S4 respectively. **(c)** Comparison between the sampled region of AMBER 99SB-IDLN trajectories (black) and the χOL3-corrected ones (orange).

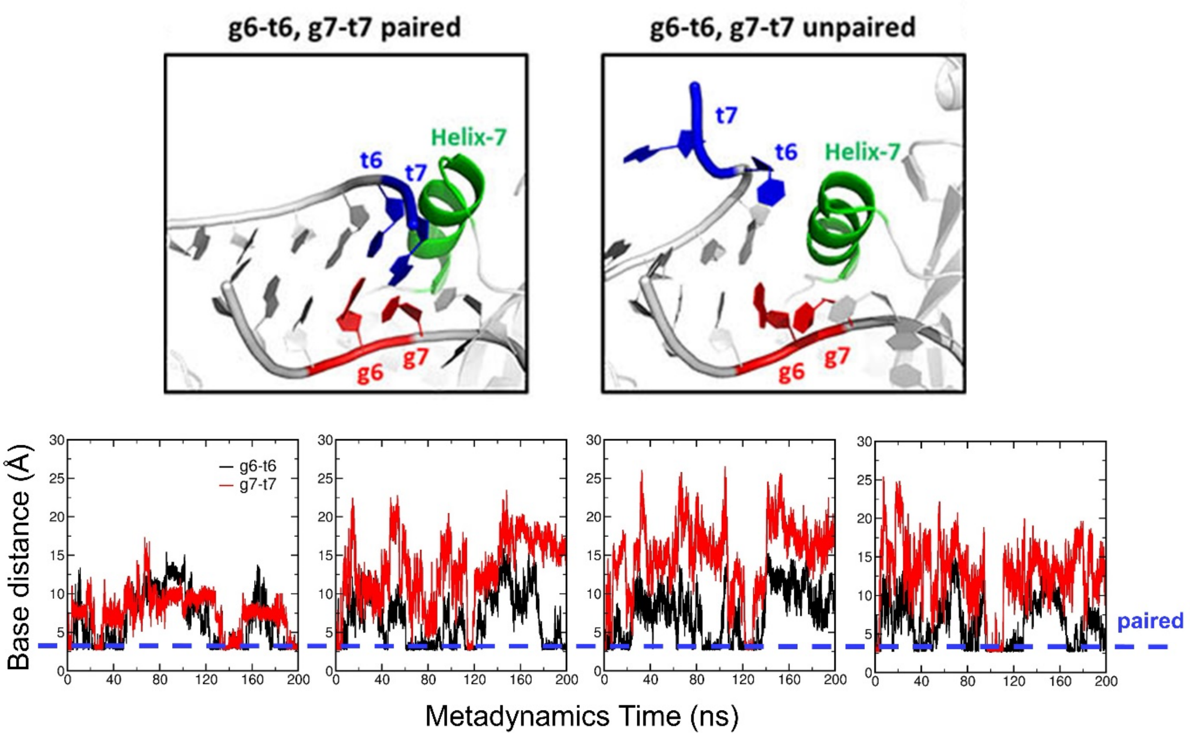

Supplementary Figure S7. Metadynamics simulations to induce un-pairing at position 6,7.

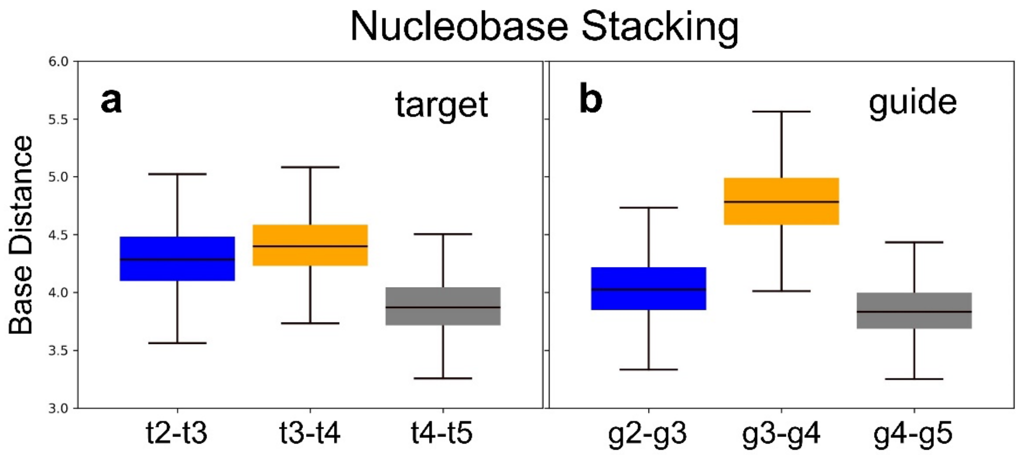

Supplementary Figure S8. **Stability of base stacking at the first half of the seed region**. Boxplot of the distance between the center of mass of the consecutive RNA nucleobases at g2-g5 for **(a)** the target RNA and **(b)** the guide RNA. Statistics was made on all data used to construct our MSMs.

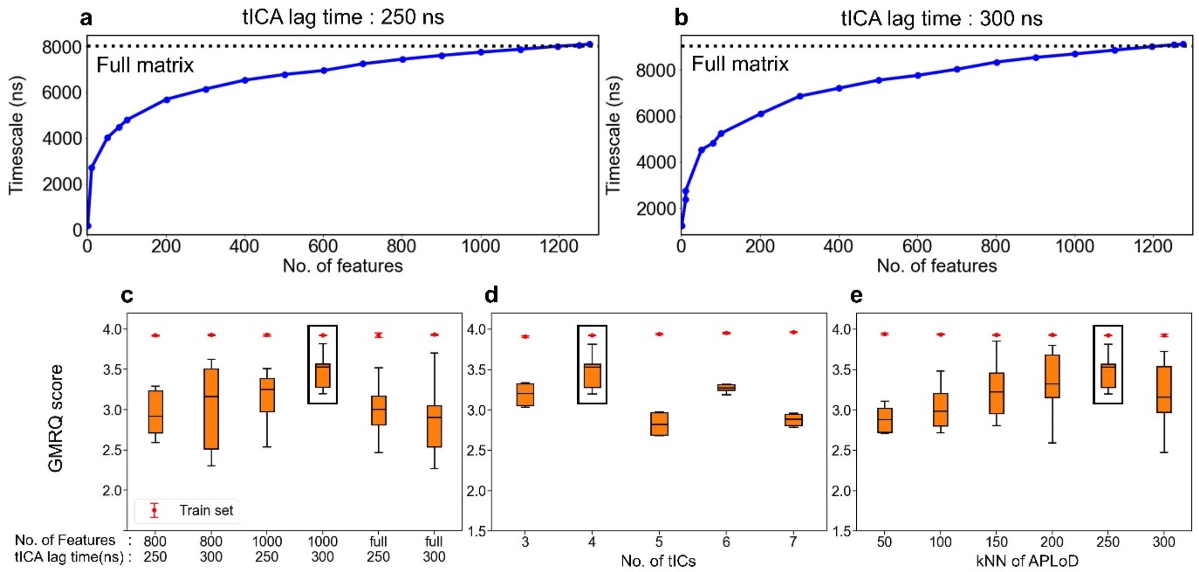

Supplementary Figure S9. Construction of the microstate MSM. The slowest tICA implied timescale is plotted as a function of number of features selected by Spectral oASIS with tICA lag time **(a)** 250ns and **(b)** 300ns. GMRQ tests to choose optimal values for **(c)** number of features and tICA lag time, **(d)** number of tICs and **(e)** k-nearest neighbors of APLoD clustering. For all the GMRQ tests, the MSM lag time is 380ns.

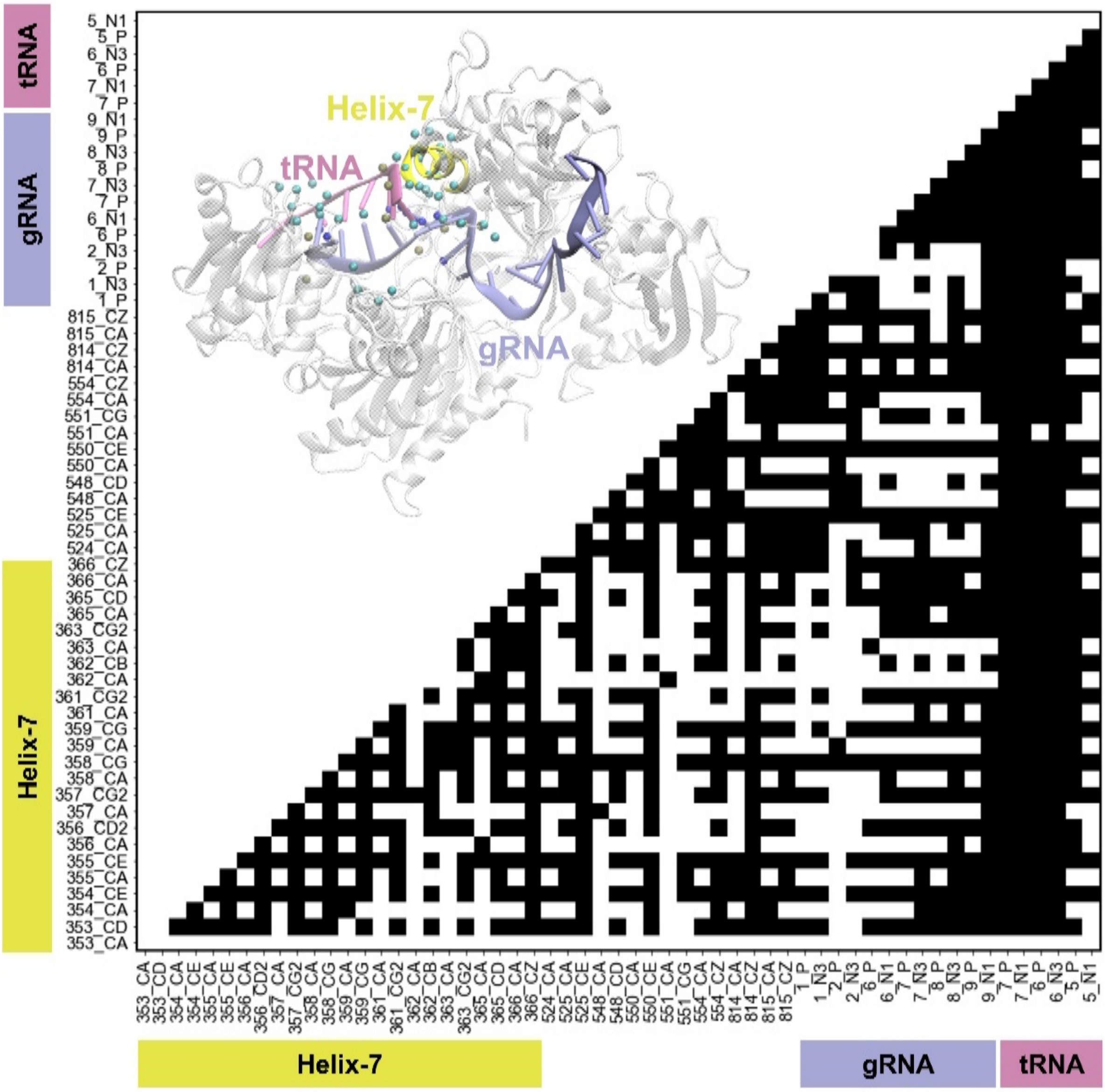

Supplementary Figure S10. The input features for tICA, set of pairwise distances between pairs of atoms selected by Spectral oASIS.

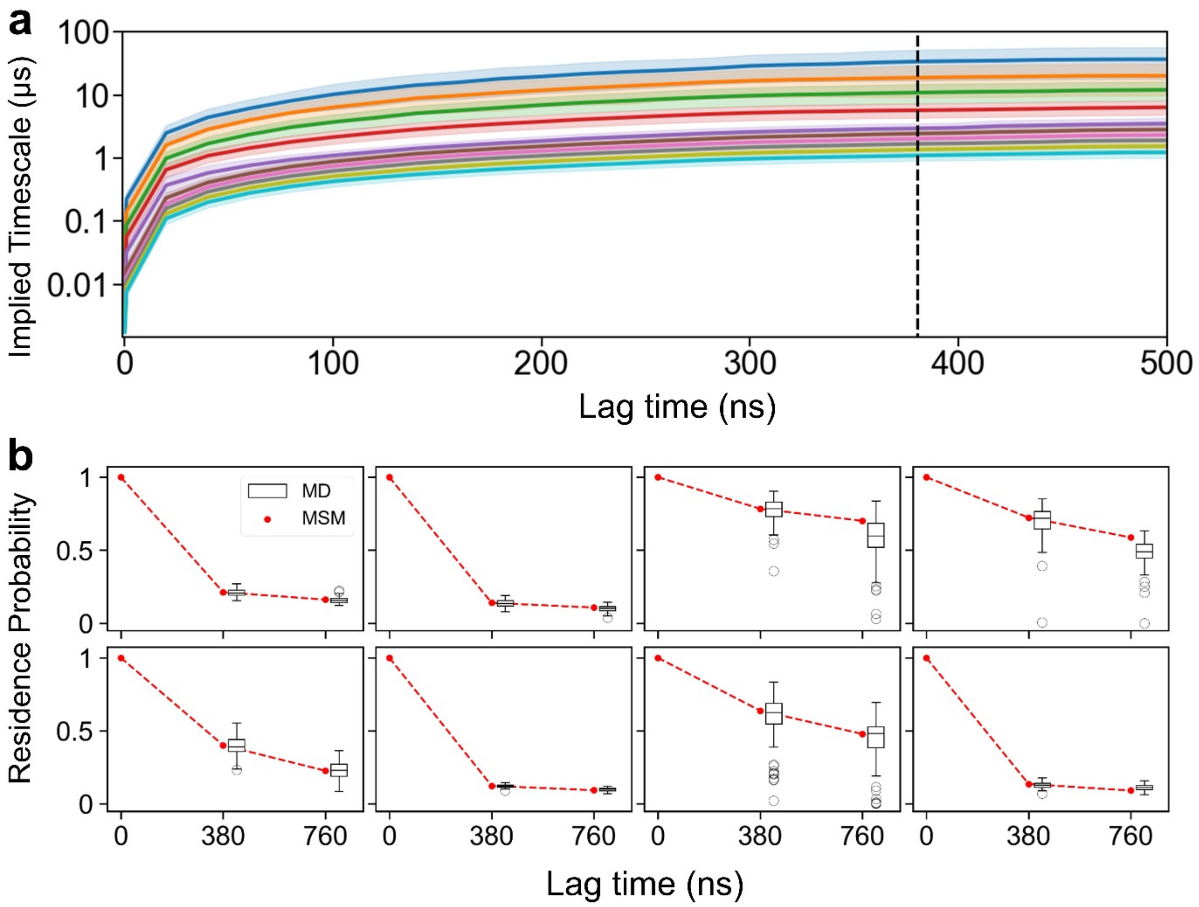

Supplementary Figure S11. Validation of the microstate MSM. (a) Implied timescale (ITS) plot of the microstate MSM. Colors of the solid lines indicate different implied timescales of the microstates MSMs. The black dashed line is the MSM lag time used to build MSM, 380 ns. **(b)** Chapman-Kolmogorov: residence probability test for the 8 most populated microstates. The error bars in the (a) and (b) are calculated by bootstrapping the independent MD trajectories 20 times.

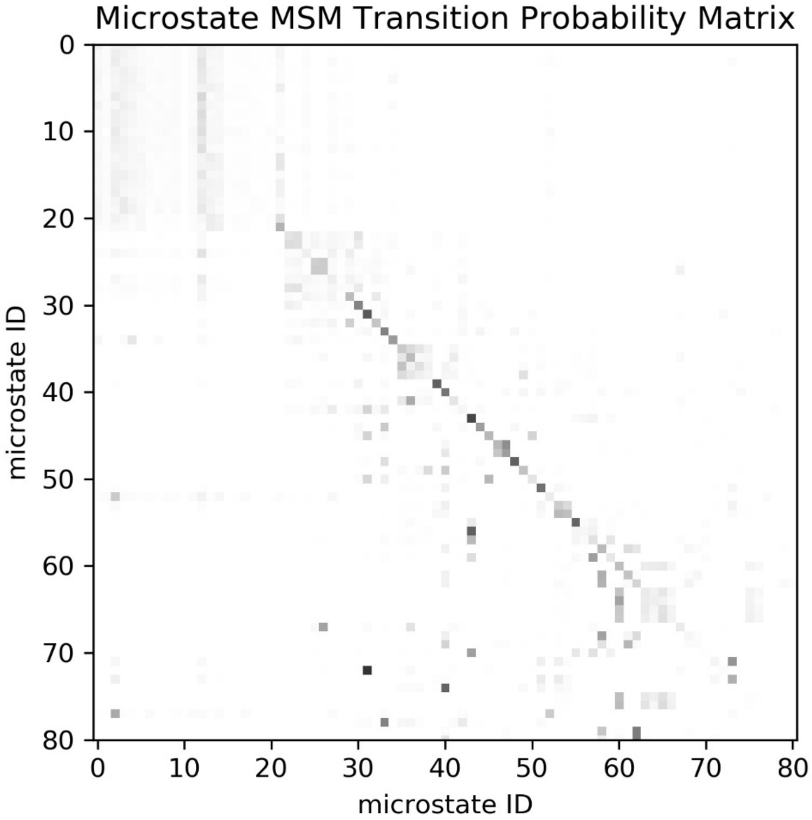

Supplementary Figure S12. The transition probability matrix of the 81-microstate MSM.

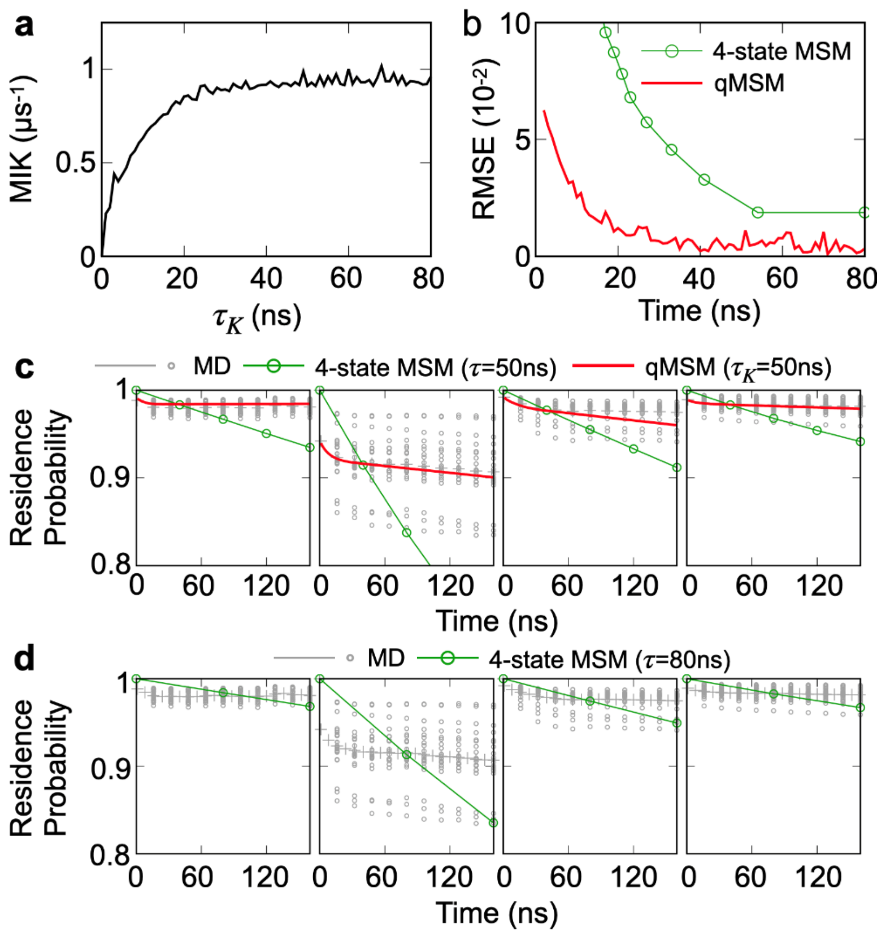

Supplementary Figure S13. Validation of the qMSM model. (a) Mean integral of the memory kernel (MIK) curve for the qMSM memory kernel as a function of the kernel cut-off time, τ_K. **(b)** Time-averaged root mean squared error (RMSE) curves for both MSM and qMSM, with respect to the benchmark TPM dynamics taken from simulation trajectories. Errors are calculated for time range 0~80ns, and MSMs/qMSMs at $\tau = 50$ns are reported. These lag times are chosen so that 160ns is divisible to τ, and the discrete time sequences of MSMs can cover the last time point 160ns. **(c)** Residence probability test of MSM and qMSM. Grey curves are directly counted from MD trajectories; green curves are the MSM built with lag time $\tau = 50$ns; and red curves are the qMSM results with $\tau_K = 50$ns. The error bar was obtained by bootstrapping the MD trajectories 20 times. **(d)** Residence probability test of MSM that built at lag time $\tau = 80$ns. Grey curves are directly counted from MD trajectories, and green curves are the MSM results.

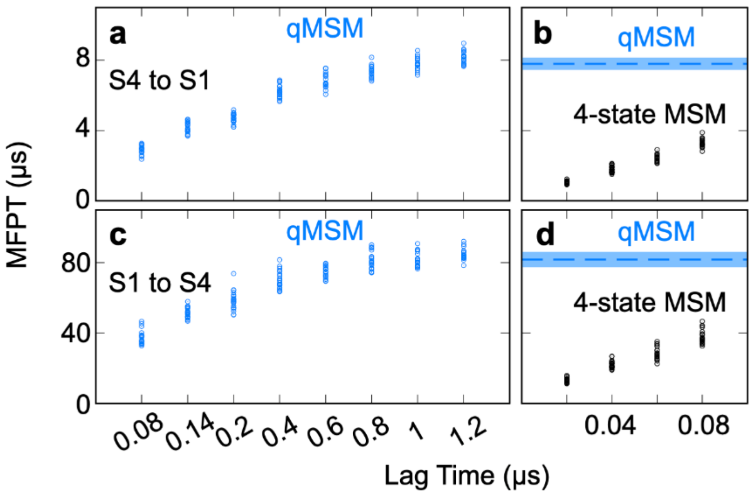

Supplementary Figure S14. Mean-first-passage-times (MFPTs) for the interconversion between the recognized state S1 and unrecognized state S4. MFPTs from S4 to S1 calculated at different lag times using **(a)** 4-state qMSM at extended lag times and **(b)** 4-state MSM. MFPTs from S1 to S4 calculated at different lag times using **(d)** 4-state qMSM at extended lag times and **(d)** 4-state MSM. The dashed line corresponds to the MFPT calculated by 4-state qMSM at lag time = 1μs. At each lag time, 20 MFPT data points were shown in circles, which were obtained by bootstrapping the MD trajectories 20 times.

Residue / functional group	Atoms
g6 base	N1
t6 base	N3
g7 base	N3
t7 base	N1
N359 (Helix-7)	Cα
Q527 (MID)	Cα
t6 phosphate	P
t7 phosphate	P
R366	CZ
R554	CZ
K550	NZ
K525	NZ
K355	NZ

Supplementary Table S1. Atoms used to define collective variables

Path ID	Fraction in total flux	Sequence of microstates
1	11.75%	43 → ***55*** → 36 →26
2	9.15%	43 → **57** → 36 → 26
3	6.74%	43 → **57** → 67 → 26
4	4.20%	43 → **57** → 35 → 36 → 31 → 26
5	3.08%	43 → **57** → 33 → 27 → 12 → 05
6	3.08%	43 → **57** → 25 → 26
7	2.87%	43 → **57** → 33 → 30 → 23 → 26
8	2.77%	43 → **57** → 26
9	2.51%	43 → 38 → 49 → 40 → 51 → 73 → 02 → 05
10	2.41%	43 → 36 → 31 → 25 → 26

Supplementary Table S2. Top 10 highest flux paths for g6-g7 recognition obtained from the microstate MSM via the TPT analysis. Microstate 43 corresponds to the geometric center of macrostate S4. Microstate 5 and 26 are used to represent macrostate S1, as they are similar with the crystal structure of the recognized state. The most frequently visited microstate (microstate 57) by the top 10 paths other than the initial (microstate 43) and final states (microstate 5 or 26) is labelled in bold. Microstate 55 in the highest flux path is labelled bold italic.

Supplementary References

1. M. Zgarbová, M. Otyepka, J. Šponer, A. Mládek, P. Banáš, T. E. Cheatham and P. Jurečka, *J. Chem. Theory Comput.*, 2011, **7**, 2886-2902.
2. P. Banáš, D. Hollas, M. Zgarbová, P. Jurečka, M. Orozco, T. E. Cheatham, J. Šponer and M. Otyepka, *J. Chem. Theory Comput.*, 2010, **6**, 3836-3849.
3. D. Tan, S. Piana, R. M. Dirks and D. E. Shaw, *Proc. Natl. Acad. Sci. U. S. A.*, 2018, **115**, E1346.
4. A. Laio and M. Parrinello, *Proc. Natl. Acad. Sci. U. S. A.*, 2002, **99**, 12562-12566.
5. A. Barducci, G. Bussi and M. Parrinello, *Phys. Rev. Lett.*, 2008, **100**, 020603.
6. G. A. Tribello, M. Bonomi, D. Branduardi, C. Camilloni and G. Bussi, *Comput. Phys. Commun.*, 2014, **185**, 604-613.
7. Myung H. Jo, S. Shin, S.-R. Jung, E. Kim, J.-J. Song and S. Hohng, *Mol. Cell*, 2015, **59**, 117-124.
8. S. D. Chandradoss, N. T. Schirle, M. Szczepaniak, I. J. MacRae and C. Joo, *Cell*, 2015, **162**, 96-107.
9. N. T. Schirle, J. Sheu-Gruttadauria and I. J. MacRae, *Science*, 2014, **346**, 608.
10. C. R. Schwantes and V. S. Pande, *J. Chem. Theory Comput.*, 2013, **9**, 2000-2009.
11. Y. Naritomi and S. Fuchigami, *J. Chem. Phys.*, 2013, **139**, 215102.
12. C. Blau and H. Grubmuller, *Comput. Phys. Commun.*, 2013, **184**, 2856-2859.
13. F. Litzinger, L. Boninsegna, H. Wu, F. Nüske, R. Patel, R. Baraniuk, F. Noé and C. Clementi, *J. Chem. Theory Comput.*, 2018, **14**, 2771-2783.
14. R. T. McGibbon and V. S. Pande, *J. Chem. Phys.*, 2015, **142**, 124105.
15. S. Liu, L. Zhu, F. K. Sheong, W. Wang and X. Huang, *J. Comput. Chem.*, 2017, **38**, 152-160.
16. K. A. Beauchamp, G. R. Bowman, T. J. Lane, L. Maibaum, I. S. Haque and V. S. Pande, *J. Chem. Theory Comput.*, 2011, **7**, 3412-3419.
17. S. Rblitz and M. Weber, *Advances in Data Analysis and Classification*, 2013, **7**.
18. M. K. Scherer, B. Trendelkamp-Schroer, F. Paul, G. Perez-Hernandez, M. Hoffmann, N. Plattner, C. Wehmeyer, J. H. Prinz and F. Noe, *J. Chem. Theory Comput.*, 2015, **11**, 5525-5542.
19. S. Cao, A. Montoya-Castillo, W. Wang, T. E. Markland and X. Huang, *J. Chem. Phys.*, 2020, **153**, 014105.
20. G. R. Bowman, K. a. Beauchamp, G. Boxer and V. S. Pande, *J. Chem. Phys.*, 2009, **131**, 124101.
21. J. H. Prinz, H. Wu, M. Sarich, B. Keller, M. Senne, M. Held, J. D. Chodera, C. Schutte and F. Noe, *J. Chem. Phys.*, 2011, **134**, 174105.
22. P. Metzner, C. Schütte and E. Vanden-Eijnden, *Multiscale Model. Simul.*, 2009, **7**, 1192-1219.
23. E. Weinan and E. Vanden-Eijnden, *J. Stat. Phy.*, 2006, **123**, 503-523.
