# Supplementary material for: Critical role of backbone coordination in the mRNA recognition by RNA induced silencing complex
Source: Commun Biol. 2021 Nov 30;4:1345. doi: 10.1038/s42003-021-02822-7 (PMC8632932; doi:10.1038/s42003-021-02822-7)
Supplement: Supplementary file 2 — Description of Additional Supplementary Files [file 42003_2021_2822_MOESM2_ESM.pdf]

Description of Additional Supplementary Files

File name: Supplementary Movie 1.
Description: A MD trajectory where near-complete recognition at g6-g7 is observed. Key residues for this process are highlighted in color.

File name: Supplementary Data 1.
Description: Data for plotting boxplots in Figs 2 and 3.
